# Analysis of Retinal Thickness in Patients With Chronic Diseases Using Standardized Optical Coherence Tomography Data: Database Study Based on the Radiology Common Data Model

**DOI:** 10.2196/64422

**Published:** 2025-02-21

**Authors:** ChulHyoung Park, So Hee Lee, Da Yun Lee, Seoyoon Choi, Seng Chan You, Ja Young Jeon, Sang Jun Park, Rae Woong Park

**Affiliations:** 1 Department of Biomedical Informatics, Ajou University School of Medicine, 206, Worldcup-ro, Yeongtong-gu, Suwon, 16499, Republic of Korea, 82 31-219-4471; 2 Department of Biomedical Sciences, Ajou University Graduate School of Medicine, Suwon, Republic of Korea; 3 Department of Scientific Solutions, CMIC Korea Co, Ltd, Seoul, Republic of Korea; 4 Department of Ophthalmology, Seoul National University Bundang Hospital, Seongnam, Republic of Korea; 5 Department of Biomedical Systems Informatics, Yonsei University College of Medicine, Seoul, Republic of Korea; 6 Department of Endocrinology and Metabolism, Ajou University School of Medicine, Suwon, Republic of Korea

**Keywords:** data standardization, ophthalmology, radiology, optical coherence tomography, retinal thickness

## Abstract

**Background:**

The Observational Medical Outcome Partners-Common Data Model (OMOP-CDM) is an international standard for harmonizing electronic medical record (EMR) data. However, since it does not standardize unstructured data, such as medical imaging, using this data in multi-institutional collaborative research becomes challenging. To overcome this limitation, extensions such as the Radiology Common Data Model (R-CDM) have emerged to include and standardize these data types.

**Objective:**

This work aims to demonstrate that by standardizing optical coherence tomography (OCT) data into an R-CDM format, multi-institutional collaborative studies analyzing changes in retinal thickness in patients with long-standing chronic diseases can be performed efficiently.

**Methods:**

We standardized OCT images collected from two tertiary hospitals for research purposes using the R-CDM. As a proof of concept, we conducted a comparative analysis of retinal thickness between patients who have chronic diseases and those who have not. Patients diagnosed or treated for retinal and choroidal diseases, which could affect retinal thickness, were excluded from the analysis. Using the existing OMOP-CDM at each institution, we extracted cohorts of patients with chronic diseases and control groups, performing large-scale 1:2 propensity score matching (PSM). Subsequently, we linked the OMOP-CDM and R-CDM to extract the OCT image data of these cohorts and analyzed central macular thickness (CMT) and retinal nerve fiber layer (RNFL) thickness using a linear mixed model.

**Results:**

OCT data of 261,874 images from Ajou University Medical Center (AUMC) and 475,626 images from Seoul National University Bundang Hospital (SNUBH) were standardized in the R-CDM format. The R-CDM databases established at each institution were linked with the OMOP-CDM database. Following 1:2 PSM, the type 2 diabetes mellitus (T2DM) cohort included 957 patients, and the control cohort had 1603 patients. During the follow-up period, significant reductions in CMT were observed in the T2DM cohorts at AUMC (*P*=.04) and SNUBH (*P*=.007), without significant changes in RNFL thickness (AUMC: *P*=.56; SNUBH: *P*=.39). Notably, a significant reduction in CMT during the follow-up was observed only at AUMC in the hypertension cohort, compared to the control group (*P*=.04); no other significant differences in retinal thickness were found in the remaining analyses.

**Conclusions:**

The significance of our study lies in demonstrating the efficiency of multi-institutional collaborative research that simultaneously uses clinical data and medical imaging data by leveraging the OMOP-CDM for standardizing EMR data and the R-CDM for standardizing medical imaging data.

## Introduction

The Observational Medical Outcomes Partnership-Common Data Model (OMOP-CDM) is an internationally standardized model designed to harmonize medical data across various health care institutions [1]. Its primary goal is to facilitate efficient, large-scale, multi-institutional collaborative research. Currently, a federated database developed collaboratively by researchers from over 80 countries contains data from 12% of the global population [2].

The OMOP-CDM is a model that standardizes structured clinical data such as diagnoses, prescriptions, and procedures. However, unstructured data, such as genomic and imaging data, often fall outside the standardization scope of the current OMOP-CDM, presenting challenges for use in multi-institutional collaborative research. To address this limitation, extended models based on the OMOP-CDM are being actively developed [3,4]. One such model used in this study, known as the Radiology Common Data Model (R-CDM), standardizes imaging data, enabling the efficient integration of multi-institutional imaging and clinical data to enhance research capabilities [5].

Optical coherence tomography (OCT) captures detailed images of the eye’s internal structure, including parameters such as retinal thickness. Studies using OCT data have explored relationships between retinal thickness and various factors including age, hypertension, type 2 diabetes mellitus (T2DM), and vitamin D deficiency [6-8]. However, these investigations have generally been limited by their confinement to single medical institutions with small patient cohorts, due to the simultaneous requirement for clinical and imaging data. To address these constraints, this study aims to standardize multi-institutional OCT data using the R-CDM, thereby facilitating research that integrates clinical and imaging data across multiple institutions.

## Methods

### Data Sources

This study used clinical and imaging data from two of the leading tertiary hospitals in South Korea: Ajou University Medical Center (AUMC) and Seoul National University Bundang Hospital (SNUBH). For clinical data, standardized OMOP-CDM databases were used. The AUMC OMOP-CDM database included standardized electronic medical record (EMR) data spanning from 1994 to February 2023, with records from 2,752,765 patients. The SNUBH OMOP-CDM database contained standardized EMR data from April 2003 to 2021, covering 2,017,421 patients. For imaging data, we had authorization to use selected OCT data collected with specific medical devices. OCT data were typically stored on the OCT device itself and were not automatically integrated into the EMR or picture archiving communication systems. To use these data for research purposes, the stored images had to be manually collected and converted into a usable format. Due to these limitations, OCT data were collected from all devices available at the participating institutions. Rather than selectively choosing specific devices or time periods, an inclusive approach was adopted to address potential selection bias. As a result, AUMC provided access to OCT data captured with Zeiss medical devices from 2013 to April 2022, while SNUBH granted access to OCT data obtained with Heidelberg medical devices from July 2006 to August 2019, for research purposes. This study was conducted per the Strengthening the Reporting of Observational Studies in Epidemiology (STROBE) guidelines to ensure transparency [9].

### Ethical Considerations

We obtained ethical approval from the Institutional Review Board of AUMC (approval number: AJOUIRB-DB-2024‐341) and SNUBH (approval number: SNUBH-EX-2024‐123). This study was conducted with an exemption for informed consent, as there were no direct benefits or risks to participants arising from their involvement in the study. All data were anonymized to ensure participant confidentiality and privacy. No identifying information was retained during the data analysis process.

### Constructing an R-CDM–Standardized OCT Database

Although the clinical data in this study had been standardized according to the OMOP-CDM and were ready for immediate application in multi-institutional research, the OCT imaging data still needed to be standardized to the R-CDM format to be efficiently used in research. The R-CDM standardized medical data through two distinct tables: the radiology occurrence table and the radiology image table. The radiology occurrence table cataloged each imaging event, detailing which patient used which company’s equipment, the type of imaging performed, and the date on which it was performed. The radiology image table organized information about each image, including the type, file path, and resolution. These tables were interconnected through a “study_id” that served as the primary key, allowing researchers to uniformly extract specific types of image data from imaging events.

**Figure 1.**
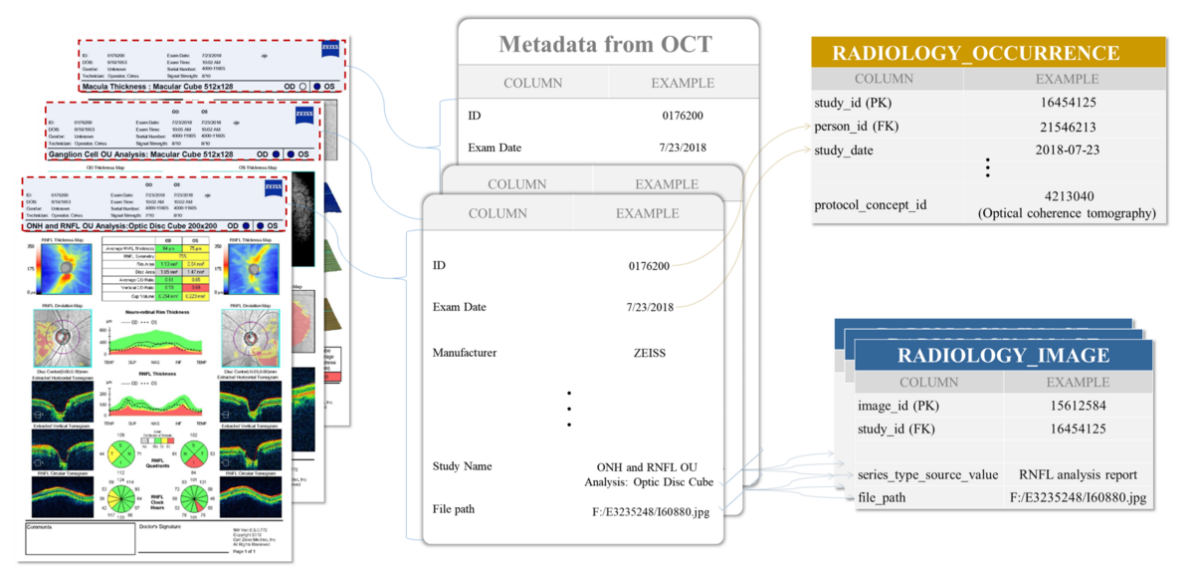
Standardizing OCT imaging data in the format of Radiology Common Data Model. OCT: optical coherence tomography.

Consequently, a process was necessary to extract metadata within the OCT data using optical character recognition (OCR) techniques and load it into the R-CDM format. For OCT data captured using Zeiss medical devices at AUMC, the sections of OCT images documenting retinal thickness were cropped, and data extraction was performed using the Python Tesseract package. OCT data from SNUBH, taken with Heidelberg medical devices, were processed using a proprietary OCR machine learning model [10]. Only high-quality OCT scans (signal strength ≥7) were used in this study. Figure 1 illustrates the process of standardizing OCT imaging data into the R-CDM format.

### Study Design With the OMOP-CDM

**Figure 2.**
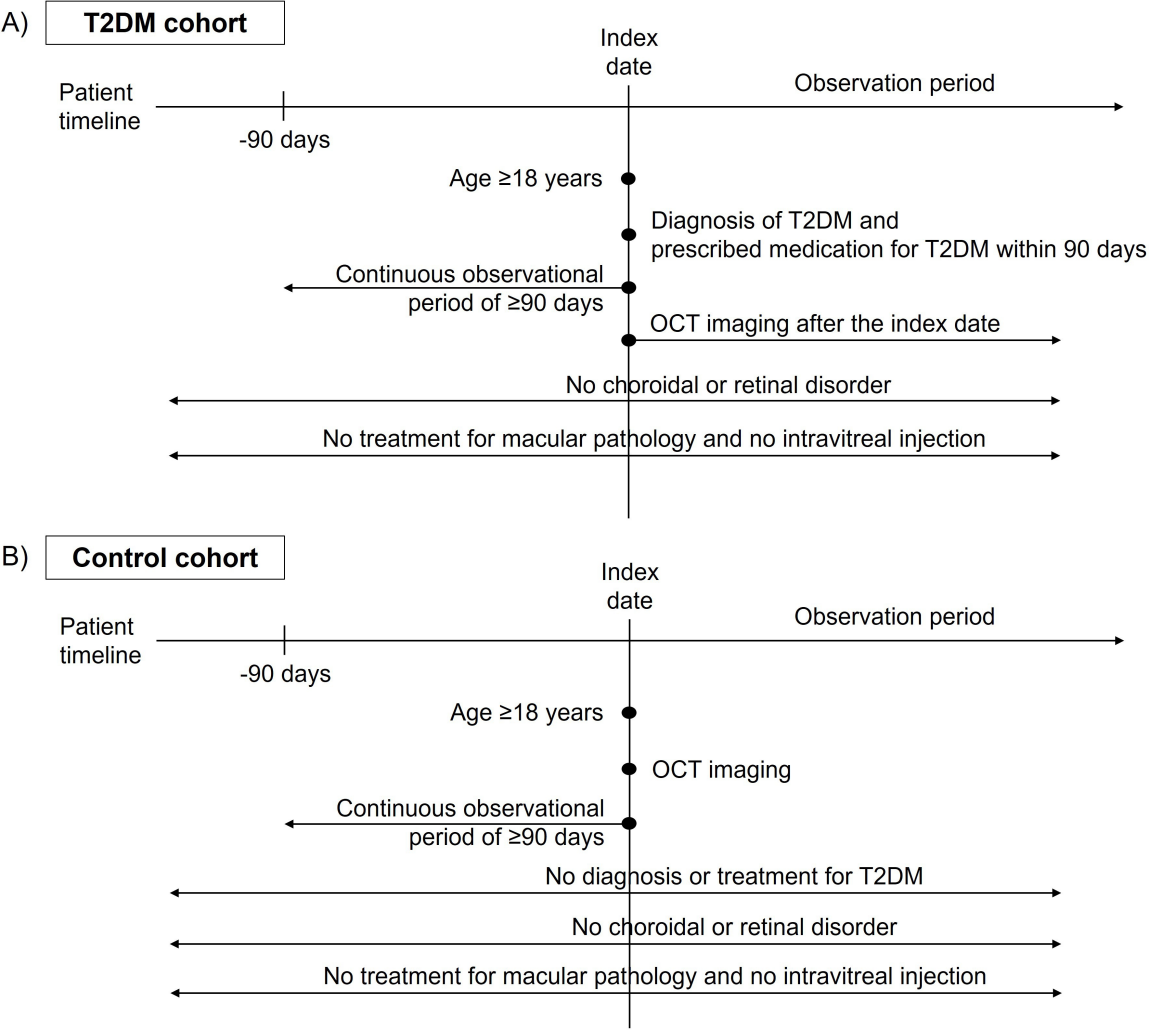
Cohort definitions for T2DM and control groups. (**A**) Patients with T2DM, excluding those diagnosed or treated for choroidal or retinal disorders, who have undergone OCT imaging. (**B**) Patients without T2DM or any choroidal or retinal disorders, who have undergone OCT imaging. OCT: optical coherence tomography. T2DM: type 2 diabetes mellitus.

In this study, we performed retinal thickness comparisons between cohorts of adult patients (aged ≥18 years) with and without chronic diseases, all of whom had undergone OCT. Among the chronic diseases, we focused on T2DM and hypertension. To ensure the comprehensive inclusion of all patients with these conditions, we used the highest-level SNOMED (Systematized Nomenclature of Medicine) hierarchy codes for enrollment. First, a T2DM cohort composed of patients diagnosed with T2DM and a control cohort of patients who were neither diagnosed with nor treated for T2DM were established. Eligibility for the T2DM cohort required a T2DM diagnosis and the administration of T2DM medications within 3 months of diagnosis, with the diagnosis date set as the index date. Patients who discontinued T2DM medications for more than 90 days were considered lost to follow-up. The control cohort included adult patients who were neither diagnosed with nor treated for T2DM, and the OCT imaging date was the index date in this cohort. Patients who had not been observed in the 90 days that preceded their respective index dates were excluded from the study. Additionally, patients with any recorded diagnosis or treatment for retinal or choroidal diseases that could affect the retinal thickness were also excluded (Figure 2). Second, an additional cohort of patients diagnosed with hypertension and a control group of patients who were neither diagnosed with nor treated for hypertension, who also had records of undergoing OCT imaging, were established in Figure E1 in Multimedia Appendix 1. Patients who discontinued hypertension medications for more than 90 days were considered lost to follow-up.

### Statistical Analysis 1: Large-Scale Propensity Score Matching for Characteristics Between Chronic Disease Cohort and Control Cohort

The propensity score matching (PSM) method was used to adjust for potential biases arising from confounding variables between the cohorts of patients with chronic diseases and the control cohorts. Propensity scores were estimated through a logistic regression model, and matching was performed using a nearest-neighbor approach within a 0.2-SD caliper on the logit scale, as recommended by Austin [11]. The balance of covariates was assessed using the absolute standardized mean difference (aSMD), with covariates including age categorized into 5-year bands, sex, and all condition diagnoses recorded in the year preceding the index date. Various matching ratios (1:1, 1:2, and 1:4) were explored for the sensitivity analysis.

### Interworking of the R-CDM and OMOP-CDM

During the standardization of OCT imaging data to the R-CDM, patient numbers used in the hospital were pseudonymized and replaced with research patient IDs for use in the OMOP-CDM database. This transformation not only eliminated the risk of personal information leakage when conducting research with imaging data but also ensured seamless integration between the OMOP-CDM and R-CDM. Following these processes, R-CDM-based OCT databases were established in both institutions.

**Figure 3.**
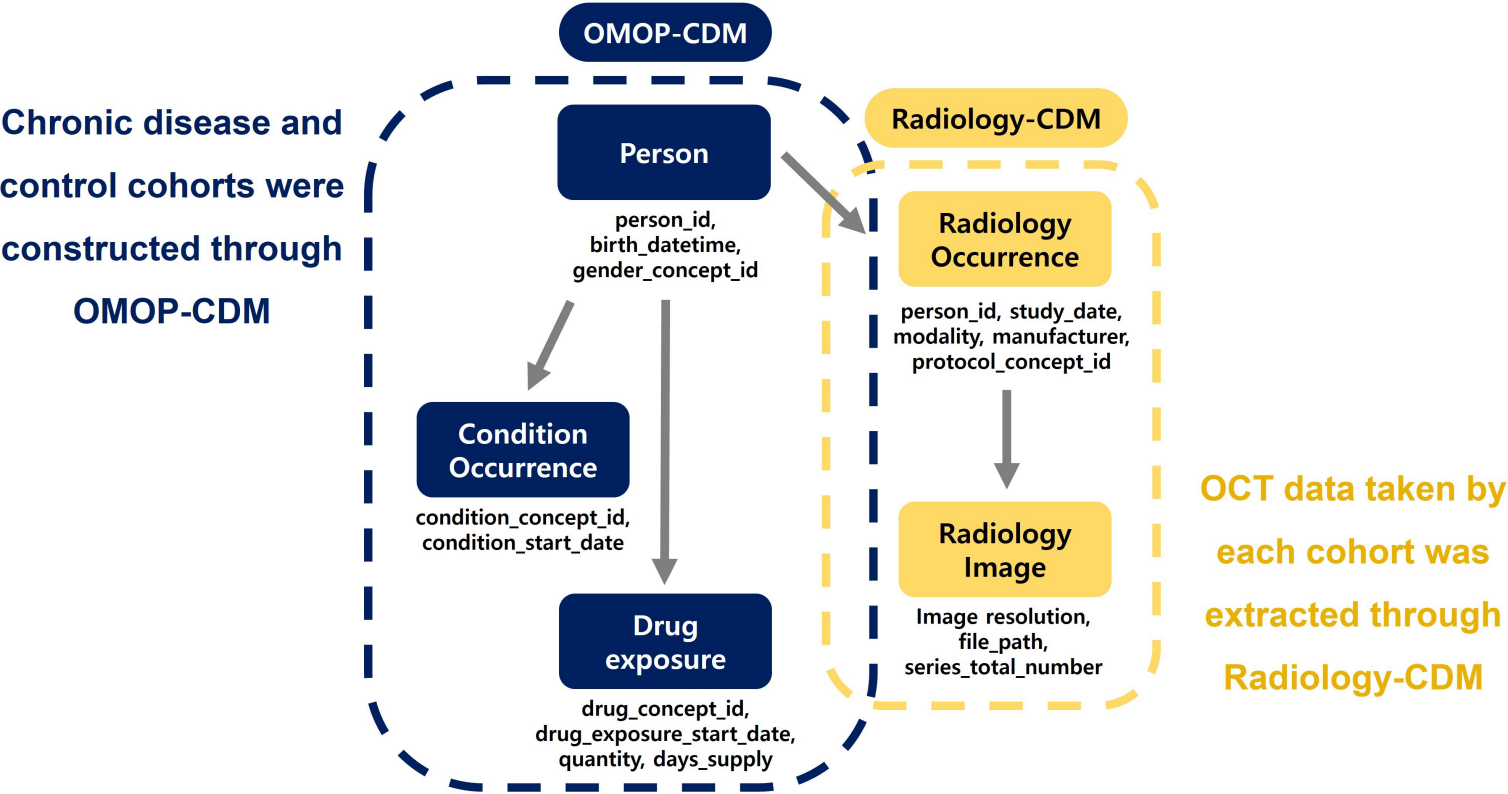
Schematic illustration of the integration between the OMOP-CDM and Radiology-CDM for extracting optical coherence tomography data from chronic disease and control cohorts. CDM: Common Data Model; OMOP-CDM: Observational Medical Outcome Partners-Common Data Model.

By linking the OMOP-CDM and R-CDM, an environment was established that enabled the extraction of specific medical imaging data from designated patient cohorts. The hypertensive, diabetic, and control cohorts were easily constructed using ATLAS, an open-source software provided by the Observational Health Data Sciences and Informatics (OHDSI) group, which manages OMOP-CDM development [12]. Once these cohorts were established, the OCT imaging data collected from each cohort were uniformly extracted using the R-CDM (Figure 3). In this study, all OCT data taken after the index date of each patient cohort were used for analyses.

### Extracting Retinal Thickness Values From OCT Data Using OCR Techniques

While the R-CDM database included information that was useful for describing and categorizing images, it lacked the pixel data from each image. Therefore, to extract recorded retinal thickness values from OCT data via the R-CDM, an additional OCR technology was required. The same OCR techniques previously used to extract metadata from OCT images for the R-CDM transformation were used. However, retinal thickness data within the OCT imaging data captured using Zeiss equipment at AUMC were recorded at inconsistent locations and centered on backgrounds of various colors, significantly reducing OCR accuracy. Initially, the process involved cropping the segments containing information and performing OCR using the Tesseract package (Python), consistent with the existing methods. Thereafter, all extracted data were manually reviewed by a single researcher. Through this method, retinal nerve fiber layer (RNFL) thickness and central macular thickness (CMT) were successfully extracted from OCT imaging data of chronic disease cohorts and control cohorts from the two institutions.

### Statistical Analysis 2: Retinal Thickness Analysis Using Mixed-Effects Regression Analysis

We used the mixed-effects regression analysis to examine the CMT and RNFL thickness across the chronic disease and control cohorts. This analysis was crucial for determining if the differences in retinal thickness between the two groups were statistically significant at multiple time points and whether the changes in retinal thickness over time were significant. Furthermore, the model was specifically designed to adjust for confounding factors that could influence the retinal thickness, allowing for the independent analysis of the effects of the treatment duration on the former. Additionally, the model accommodated data from repeated measurements taken from the same individuals. The model we designed for this study was adjusted for age and sex and incorporated patient study ID as a random effect for repeated measures.

## Results

### Composition of R-CDM–Standardized OCT Data

**Figure 4.**
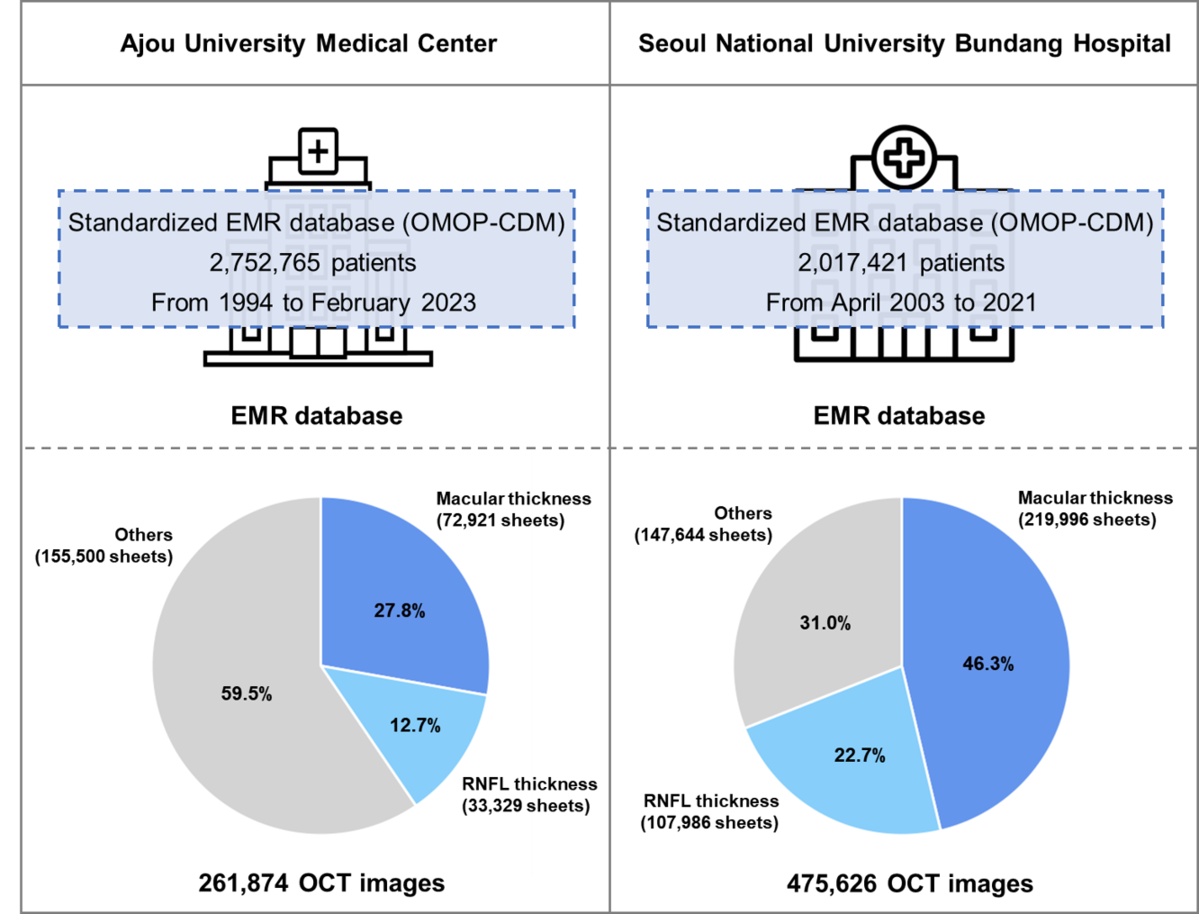
Clinical and medical imaging databases of Ajou University Medical Center and Seoul National University Bundang Hospital used in this study. EMR: electronic medical record; OCT: optical coherence tomography; OMOP-CDM: Observational Medical Outcome Partners-Common Data Model; RNFL: retinal nerve fiber layer.

In this study, we used CDM databases from two medical institutions along with an R-CDM–standardized OCT database. The OCT data from AUMC and SNUBH, standardized in the R-CDM format, consisted of data from 261,874 and 475,626 patients, respectively. The specific composition of the data is presented in Figure 4. For our analysis, a total of 292,917 OCT images of the macular thickness and 141,314 OCT images of the RNFL thickness were used.

### Study Population

**Figure 5.**
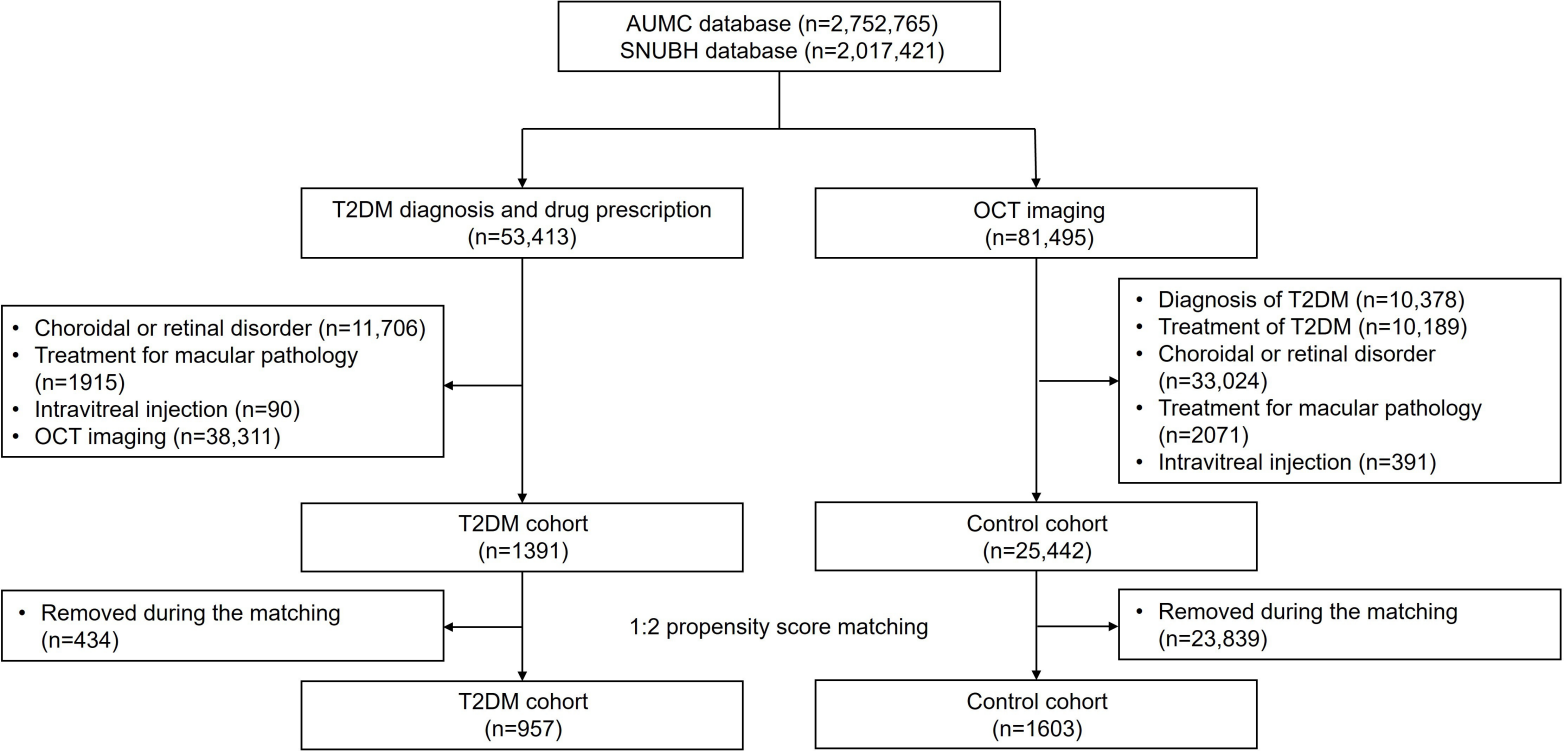
Flowchart of the study population in the comparison of T2DM and control cohorts. AUMC: Ajou University Medical Center; OCT: optical coherence tomography; SNUBH: Seoul National University Bundang Hospital; T2DM: type 2 diabetes mellitus.

Figure 5 illustrates the patient selection process across two tertiary hospitals in South Korea, using the AUMC and SNUBH databases. Within the T2DM cohort, from a total of 53,413 patients with diabetes and 1391 individuals who had undergone OCT imaging and were not diagnosed with or treated for any retinal diseases were selected. In the control cohort, out of 116,637 patients who underwent OCT imaging, 25,442 with no history of diabetes or retinal disorders were chosen. Following 1:2 PSM, 957 patients from the T2DM cohort and 1,603 from the control cohort were matched. After PSM, the median age group for the T2DM and control cohorts was 60‐64 years. Additionally, females accounted for 44.6% (427/957) and 43.9% (703/1603) of patients in the T2DM and control cohorts, respectively.

Table 1 presents the baseline characteristics, including patient demographics and comorbidities, along with aSMDs before and after PSM at AUMC. Before PSM, higher prevalences of chronic conditions such as chronic liver disease, hyperlipidemia, and obesity, as well as cardiovascular diseases including coronary arteriosclerosis and peripheral vascular disease were observed in the T2DM group (all aSMDs>0.1). After matching, age, sex, and comorbidities were well-matched (all aSMDs<0.1). This trend was consistent with findings at SNUBH, as detailed in Multimedia Appendix 1. Additionally, the baseline characteristics before and after 1:1 and 1:4 PSM for the T2DM and control cohorts and the results for the hypertension versus control cohort are outlined in Tables E1 and E2 in Multimedia Appendix 1.

**Table 1.** Comparative analysis of baseline characteristics and comorbidity profiles between the T2DM and control cohorts before and after the 1:2 PSM at Ajou University Medical Center.

Characteristics	Before PSM^a^ matching			After PSM matching		
	T2DM^b^ (n=967)	Control (n=11,896)	aSMD^c^	T2DM (n=633)	Control (n=1045)	aSMD
Age (years), n (%)						
15‐19	4 (0.4)	190 (1.6)	0.12	4 (0.6)	7 (0.7)	0.01
20‐24	3 (0.3)	428 (3.6)	0.24	3 (0.5)	10 (1)	0.04
25‐29	10 (1)	393 (3.3)	0.15	8 (1.3)	20 (1.9)	0.04
30‐34	17 (1.8)	571 (4.8)	0.17	11 (1.7)	26 (2.5)	0.04
35‐39	38 (3.9)	904 (7.6)	0.16	25 (3.9)	33 (3.2)	0.05
40‐44	56 (5.8)	1083 (9.1)	0.12	40 (6.3)	72 (6.9)	0.00
45‐49	84 (8.7)	1190 (10)	0.05	60 (9.5)	96 (9.2)	0.02
50‐54	136 (14.1)	1249 (10.5)	0.11	91 (14.4)	177 (16.9)	0.06
55‐59	132 (13.7)	1332 (11.2)	0.08	90 (14.2)	140 (13.4)	0.02
60‐64	149 (15.4)	1249 (10.5)	0.15	92 (14.5)	143 (13.7)	0.01
65‐69	113 (11.7)	1130 (9.5)	0.07	65 (10.3)	112 (10.7)	0.03
70‐74	111 (11.5)	904 (7.6)	0.13	72 (11.4)	98 (9.4)	0.05
75‐79	81 (8.4)	690 (5.8)	0.10	49 (7.7)	66 (6.3)	0.04
80‐84	27 (2.8)	393 (3.3)	0.03	20 (3.2)	28 (2.7)	0.02
85‐89	6 (0.6)	167 (1.4)	0.08	3 (0.5)	11 (1.1)	0.07
Sex, n (%)						
Female	461 (47.7)	6186 (52)	0.09	301 (47.6)	481 (46)	0.05
Male	506 (52.3)	5710 (48)	0.03	332 (52.4)	564 (54)	0.03
Medical history (general), n (%)						
Acute respiratory disease	19 (2)	83 (0.7)	0.11	7 (1.1)	11 (1.1)	0.00
Chronic liver disease	29 (3)	48 (0.4)	0.20	14 (2.2)	17 (1.6)	0.02
Chronic obstructive lung disease	9 (0.9)	59 (0.5)	0.05	4 (0.6)	11 (1.1)	0.07
Dementia	9 (0.9)	71 (0.6)	0.04	7 (1.1)	10 (1)	0.02
Depressive disorder	20 (2.1)	131 (1.1)	0.08	9 (1.4)	25 (2.4)	0.06
Gastroesophageal reflux disease	35 (3.6)	333 (2.8)	0.04	22 (3.5)	41 (3.9)	0.04
Gastrointestinal hemorrhage	10 (1)	48 (0.4)	0.07	8 (1.3)	15 (1.4)	0.01
HIV infection	2 (0.2)	12 (0.1)	0.02	1 (0.2)	3 (0.3)	0.02
Hyperlipidemia	96 (9.9)	333 (2.8)	0.29	40 (6.3)	60 (5.7)	0.00
Lesion of liver	39 (4)	131 (1.1)	0.19	21 (3.3)	31 (3)	0.01
Obesity	17 (1.8)	48 (0.4)	0.13	11 (1.7)	10 (1)	0.06
Osteoarthritis	18 (1.9)	131 (1.1)	0.07	11 (1.7)	15 (1.4)	0.03
Urinary tract infectious disease	15 (1.6)	36 (0.3)	0.13	10 (1.6)	9 (0.9)	0.06
Viral hepatitis C	5 (0.5)	12 (0.1)	0.08	1 (0.2)	3 (0.3)	0.03
Medical history (cardiovascular disease)						
Cerebrovascular disease	31 (3.2)	202 (1.7)	0.10	18 (2.8)	39 (3.7)	0.07
Coronary arteriosclerosis	120 (12.4)	202 (1.7)	0.43	54 (8.5)	85 (8.1)	0.07
Heart disease	201 (20.8)	535 (4.5)	0.51	99 (15.6)	150 (14.4)	0.06
Peripheral vascular disease	37 (3.8)	36 (0.3)	0.25	7 (1.1)	13 (1.2)	0.03
Pulmonary embolism	3 (0.3)	12 (0.1)	0.04	3 (0.5)	6 (0.6)	0.04
Medical history (neoplasm), n (%)						
Hematologic neoplasm	24 (2.5)	107 (0.9)	0.12	14 (2.2)	29 (2.8)	0.07
Malignant neoplastic disease	92 (9.5)	630 (5.3)	0.16	54 (8.5)	103 (9.9)	0.09
Malignant tumor of breast	8 (0.8)	95 (0.8)	0.00	4 (0.6)	11 (1.1)	0.05
Malignant tumor of colon	4 (0.4)	36 (0.3)	0.01	2 (0.3)	5 (0.5)	0.04
Malignant tumor of lung	4 (0.4)	59 (0.5)	0.01	4 (0.6)	10 (1)	0.05
Primary malignant neoplasm of prostate	5 (0.5)	48 (0.4)	0.02	5 (0.8)	11 (1.1)	0.07

a PSM: propensity score matching.

b T2DM: type 2 diabetes mellitus.

c aSMD: absolute standardized mean difference.

### Clinical Outcomes

**Figure 6.**
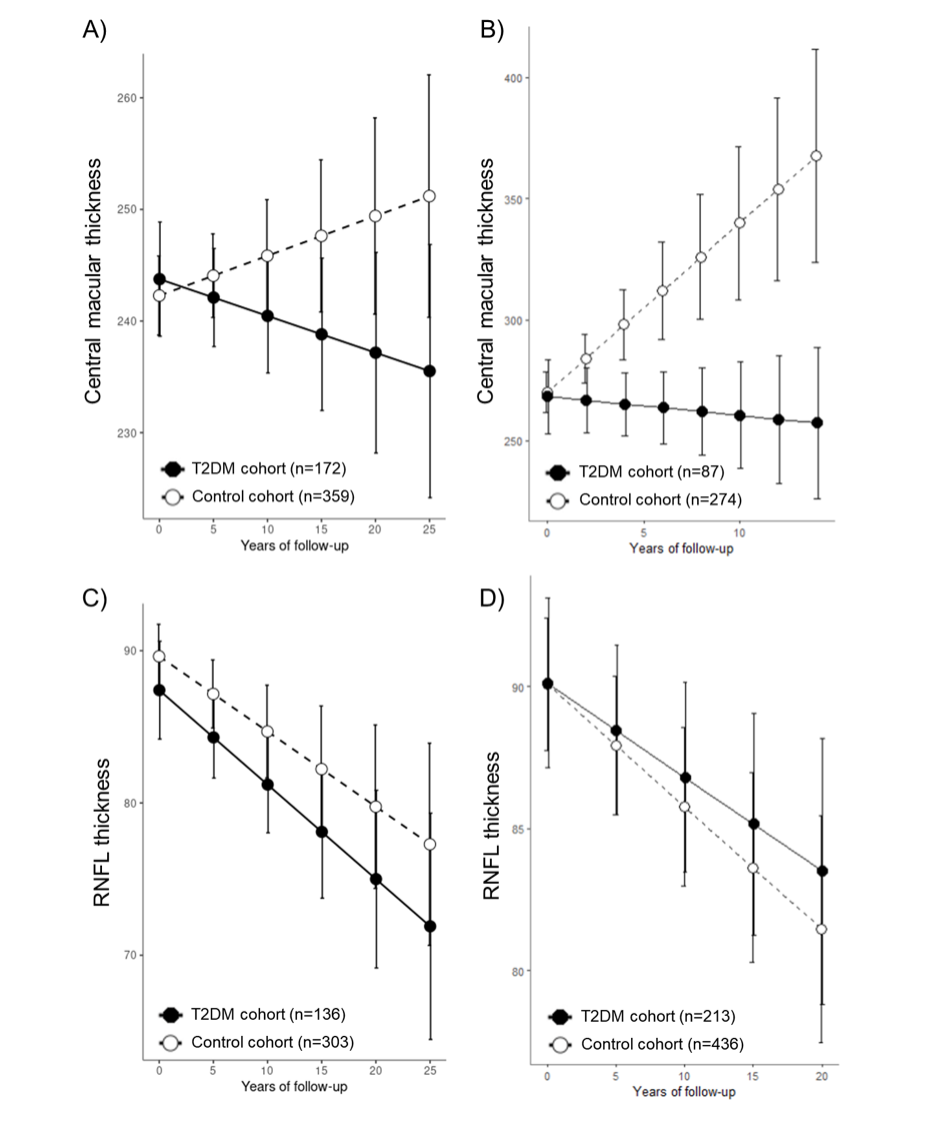
Longitudinal analysis of laboratory values using a linear mixed effects model after 1:2 propensity score matching of T2DM and control cohorts. (**A**) Central macular thickness at Ajou University Medical Center. (**B**) Central macular thickness at Seoul National University Bundang Hospital. (**C**) RNFL thickness at Ajou University Medical Center. (**D**) RNFL thickness at Seoul National University Bundang Hospital. RNFL: retinal nerve fiber layer; T2DM: type 2 diabetes mellitus.

The median follow-up period was 807 (IQR 215-2523) days (4105 person-years) in the T2DM cohort and 966 (IQR 265-2946) days (4641 person-years) in the control cohort. In a longitudinal study using a mixed-effects regression, we investigated differences in retinal thickness between the T2DM and control cohorts (Figure 6). Throughout the follow-up period at both AUMC and SNUBH, the CMT significantly decreased in the T2DM cohort compared with the control cohort (*P*=.04 and *P*=.007, respectively; detailed in Tables E3-4 and E4-4 in Multimedia Appendix 1). Initially, there were no significant differences in CMT between the T2DM and control cohorts. However, significant reductions in CMT in the T2DM cohort, compared with the control group, began from the 15th year of follow-up at AUMC and the 5th year at SNUBH (Tables E3-5 and E4-5 in Multimedia Appendix 1). Conversely, the RNFL thickness analysis revealed no significant changes in the T2DM and control cohorts during the follow-up period, and there were no significant differences between them at any time point. These findings were consistent across both institutions.

For retinal thickness comparisons between the hypertension and control cohorts during the follow-up period, a decreasing trend in CMT in the hypertension cohort compared with the control cohort was observed only at AUMC (*P*=.04), while no such trend was noted at SNUBH (*P*=.56), as detailed in Tables E3-4 and E4-4 in Multimedia Appendix 1. Additionally, the analysis of RNFL thickness showed no significant differences between the cohorts.

For the sensitivity analysis, chronic disease and control cohorts were matched in 1:1 and 1:4 ratios to analyze the CMT and RNFL thickness, with results detailed in Tables E3 and E4 and Figures E4 and E5 in Multimedia Appendix 1. The analysis of CMT between the T2DM and control cohorts revealed that, except for one outcome (1:1 at SNUBH; *P*=.24), all other results were consistent with the main findings, showing a significant decrease in CMT in the T2DM cohort throughout the follow-up duration. Additionally, while results at AUMC indicated a marginally significant decrease in CMT in the hypertension cohort compared with the control cohort during the follow-up period (all *P*<0.1), no significant differences were observed at SNUBH.

## Discussion

### Principal Results

In this study, OCT imaging data collected from two tertiary hospitals for research purposes were standardized into the R-CDM format. We developed an environment that integrates this standardized OCT database with the previously established OMOP-CDM database at each medical institution, allowing for the systematic extraction of specific imaging data types captured by patient cohorts built with the OMOP-CDM. As a proof of concept, we assessed the changes in retinal thickness in patients with chronic diseases over an extended period. Notably, we observed a significant reduction in the CMT among patients who had been receiving long-term treatment for T2DM at both institutions. This research is believed to be the first global study to simultaneously use OCT data and clinical data, demonstrating that the application of the R-CDM can significantly enhance the efficiency of multicenter collaborative research that integrates clinical and imaging data.

The R-CDM used in this study for OCT data standardization represents the first model to standardize medical imaging data based on the OMOP-CDM. It is available as an open-source software program on the official OHDSI GitHub site and was published in a 2022 paper [5] that elaborates on the structural design and terminology system of the R-CDM, and as a proof of concept, it standardizes 90 million sets of medical imaging data collected for research purposes from AUMC. Both domestically and internationally, there have been instances in which the R-CDM has been used to standardize imaging data and facilitate research with these standardized datasets. For instance, Wonkwang University Hospital established an R-CDM database of standardized abdominal computed tomography (CT) data for 123 patients with cirrhosis and 123 control patients, using these data to develop a predictive model for cirrhosis [13]. Additionally, the paper introduced a web-based management system enabling researchers to search for and download standardized R-CDM datasets, enhancing the accessibility and utility of the R-CDM. Furthermore, a study by Lee et al [14] used clinical data, magnetic resonance imaging (MRI) data, and clinical notes simultaneously to develop a multimodal deep learning model predicting treatment-resistant depression, using AUMC’s R-CDM database to extract MRI data that was suitable for study conditions. Additionally, within the European Health Data & Evidence Network project, the R-CDM was used to standardize brain CT and MRI data. This standardization successfully integrated quantitative data extracted from brain imaging studies by Icobrain’s AI model [15]. Therefore, the R-CDM supports global researchers in efficiently leveraging their medical imaging data for advanced studies.

In addition to the R-CDM used in this study, various other efforts exist to standardize medical imaging data. Rubin et al [16] proposed common data elements, which standardize radiologic reports using a uniform terminology and structure. Although common data elements effectively standardize data produced in radiology, they encounter difficulties when integrating with standardized clinical databases like the OMOP-CDM. Furthermore, there are two additional efforts to standardize medical imaging data based on the OMOP-CDM. The first is the medical imaging common data model (MI-CDM) developed by the Foundation of Research and Technology Hellas [17], and the second is the MI-CDM codeveloped by Yonsei University and Johns Hopkins University [18]. Both MI-CDM models not only facilitate the extraction of necessary data by standardizing medical imaging data, but they also design tables to accommodate features from images, thereby enhancing the efficiency of the use of these features in research. While the structures of the models differ slightly from each other, the majority of their architectures are considerably similar, and their standardization methods and principles are consistent. Researchers can select the model that best suits their immediate needs and later convert it to another model format with simple modifications to the database if needed. The multiple cases of the R-CDM and the development of enhanced models demonstrate the demand among researchers to use medical imaging data more efficiently.

In this study, we observed a significant reduction in CMT in patients receiving long-term treatment for T2DM compared to the control group, with an annual decrease of −0.69 μm (95% CI −1.34 to −0.03; *P*=.04) at AUMC and −7.78 μm (95% CI −11.84 to −3.71; *P*<.007) at SNUBH. In contrast, RNFL thickness showed no significant difference in annual changes between the two groups (AUMC: −0.13 μm, 95% CI −0.55 to 0.30, *P*=.56; SNUBH: 0.10 μm, 95% CI −0.13 to 0.32, *P*=.39). This finding highlights the subtle but measurable structural changes that occur in the macula as a result of prolonged exposure to diabetes. However, there was no significant difference in the rate of RNFL changes over time between the two groups. These results are consistent with findings reported by Oshitari et al [19], where the CMT in patients with diabetes without diabetic retinopathy was significantly thinner compared to the control group (mean 210.7, SD 28.6 μm vs mean 195.6, SD 23.3 μm; *P*=.02). Similarly, while RNFL thickness in the group without diabetic retinopathy was thinner than the control group (mean 104.4, SD 10.9 μm vs mean 100.4, SD 13.7 μm) the difference was not statistically significant. A study conducted at the Jamaica Plain Veteran’s Affairs Medical Center further supports these findings, reporting a significant decrease in macular thickness associated with a longer duration of diabetes in the group without diabetic retinopathy. They observed a significant negative correlation between retinal thickness and diabetes duration (central foveal thickness: *r*=−0.30, *P*=.003; total foveal thickness: *r*=−0.26, *P*=.012; total macular thickness: *r*=−0.26, *P*=.013) [20]. The observed reduction in macular thickness, which was significantly correlated with the duration of diabetes, is likely attributed to retinal neurodegeneration, an early event in the pathogenesis of diabetic retinopathy that precedes and contributes to diabetic microangiopathy [21].

Retinal thickness comparisons between the hypertension group and the control group revealed that only at AUMC did patients with long-term hypertension exhibit a reduction in CMT (−0.81 μm/y, 95% CI −1.57 to −0.06; *P*=.04). These findings align with prior studies conducted at Izmir Military Hospital and Samsung Medical Center, which also reported decreased macular thickness in patients with hypertension compared to control patients [22,23]. The Izmir Military Hospital reported a significantly thinner mean macular thickness in the hypertension group compared to the control group (mean 254.97, SD 21.81 μm vs mean 262.11, SD 21.05 μm; *P*=.037). Similarly, a study from Samsung Medical Center reported a significant reduction in percent thickness in patients with hypertension compared to control patients, with changes of –1.6% (95% CI −2.59% to −0.6%; *P*=.02) in the composite inner macula and −2.26% (95% CI: −3.32% to −1.19%; *P*<.01) in the composite outer macula.

Conversely, other studies have reported significantly thinner CMT and RNFL in patients with hypertension, highlighting the association between systemic hypertension and retinal microvascular abnormalities, such as retinal arteriolar narrowing and ischemic retinal disorders. These findings underscore the importance of prompt and effective management of systemic hypertension [7,24-27]. Notably, our study excluded all patients diagnosed with retinal or choroidal disorders by leveraging SNOMED codes and used large-scale PSM with thousands of covariates to control for baseline differences between groups. While these methodological approaches may account for some discrepancies with earlier studies, further large-scale research is needed to provide more conclusive evidence.

Large-scale PSM methodology, which was actively used and validated by the OHDSI community, meticulously matched baseline characteristics between the patient and control groups. This approach incorporated more covariates than reported in previous studies [28]. Additionally, while the existing literature indicates that the macular thickness is significantly higher in males than in females [29,30] and decreases with age [6,31], our study used a linear mixed model to comprehensively adjust for variables that significantly affect this parameter. Moreover, to generate more robust evidence, we conducted analyses with the same settings across multiple institutions. However, to date, most studies exploring the association between chronic diseases and retinal thickness using OCT data have been conducted at single institutions. This is primarily due to the diverse data storage formats across medical facilities, making cross-institutional research exceedingly labor-intensive. Furthermore, as our study requires both clinical and imaging data, the workload for data preprocessing increases exponentially. By leveraging the OMOP-CDM and the associated R-CDM system, we demonstrated a significant reduction in inefficiency. We anticipate that future research will achieve greater scalability and generate more generalized and impactful findings through multi-institutional analyses using the R-CDM.

### Limitations

Nevertheless, this study has the following limitations. First, while the R-CDM significantly enhances the use of medical imaging data, we encountered persistent inefficiencies due to stringent governance requirements for research approval. Although we had unrestricted access to OMOP-CDM standardized EMR databases from two institutions, the acquisition of imaging data for research purposes remained time-consuming and labor-intensive. This underscores the necessity for a more comprehensive R-CDM that standardizes all medical imaging data across multiple institutions, which is our ultimate objective. We anticipate that as the R-CDM gains widespread acceptance comparable to the OMOP-CDM, more health care institutions will adopt it for standardizing all of their medical imaging data, thereby facilitating more efficient multicenter studies incorporating imaging data. Second, due to the inherent characteristics of OCT medical devices that do not automatically integrate with the EMR or the picture archiving communication system, this study was limited to using data from OCT devices specifically approved for research purposes at each institution. Nevertheless, by securing all OCT data from specific periods and specific equipment, we minimized potential biases in data acquisition. In future investigations, we aim to incorporate all available OCT data from both institutions to enhance the robustness and generalizability of our findings. Third, despite using data from two major tertiary hospitals in Korea, the final cohort for retinal thickness analysis comprised fewer than 2000 patients. While our cohort definition and large-scale PSM technique ensured balanced comparisons, it resulted in the exclusion of a substantial number of patients. To address these limitations, we propose conducting large-scale collaborative studies involving multiple medical centers, which would ensure a sufficiently large sample size for more conclusive research.

### Conclusion

OCT imaging data were standardized into the R-CDM format from two tertiary hospitals in South Korea, and changes in the retinal thickness in patients with chronic diseases were analyzed as a proof of concept. To maximize the use of available data, mixed-effects regression analyses were conducted, revealing that patients with long-standing T2DM exhibited a significant reduction in CMT over time compared with those without the condition. The construction of R-CDM databases across multiple institutions and its integration with the OMOP-CDM has established a robust foundation for conducting efficient multi-institutional collaborative research using both clinical and imaging data, marking a significant step forward in the domain of multidisciplinary research collaborations.

## Supplementary material

10.2196/64422 Multimedia Appendix 1 Supplementary materials.

## Abbreviations

aSMD: absolute standardized mean difference
AUMC: Ajou University Medical Center
CMT: central macular thickness
CT: computed tomography
EMR: electronic medical record
MI-CDM: Medical Imaging Common Data Model
MRI: magnetic resonance imaging
OCR: optical character recognition
OCT: optical coherence tomography
OHDSI: Observational Health Data Sciences and Informatics
OMOP-CDM: Observational Medical Outcome Partners-Common Data Model
PSM: propensity score matching
R-CDM: Radiology Common Data Model
RNFL: retinal nerve fiber layer
SNOMED: Systematized Nomenclature of Medicine
SNUBH: Seoul National University Bundang Hospital
STROBE: Strengthening the Reporting of Observational Studies in Epidemiology
T2DM: type 2 diabetes mellitus
