# Supplementary material for: Analysis of Retinal Thickness in Patients With Chronic Diseases Using Standardized Optical Coherence Tomography Data: Database Study Based on the Radiology Common Data Model
Source: JMIR Med Inform. 2025 Feb 21;13:e64422. doi: 10.2196/64422 (PMC11870599; doi:10.2196/64422)

**Analysis of Retinal Thickness in Patients with Chronic Diseases Using Standardized Optical Coherence Tomography Databases Based on the Radiology Common Data Model (R-CDM)**

**Supplementary Materials**

**Table E1.** Comparative analysis of baseline characteristics and comorbidity profiles between the chronic diseases and control cohorts before/after the propensity score matching at AUMC

**Table E2.** Comparative analysis of baseline characteristics and comorbidity profiles between the chronic diseases and control cohorts before/after the propensity score matching at SNUBH

**Table E3.** Mixed-effects regression analysis of OCT values and group effects at various time points (AUMC)

**Table E4.** Mixed-effects regression analysis of OCT values and group effects at various time points (SNUBH)

**Figure E1.** Cohort definitions for chronic diseases and control groups.

**Figure E2.** Flowchart of the study population comparing T2DM and control cohorts across hospitals

**Figure E3.** Flowchart of the study population comparing HTN and control cohorts

**Figure E4.** Longitudinal analysis of laboratory values using a linear mixed effects model of T2DM and control cohorts

**Figure E5.** Longitudinal analysis of laboratory values using a linear mixed effects model of HTN and control cohorts **Table E1-1.** Comparative analysis of baseline characteristics and comorbidity profiles between the type 2 diabetes and control cohorts before/after the 1:1 propensity score matching at AUMC

| **Characteristics** | **Before PS matching** | | | **After PS matching** | | |
| --- | --- | --- | --- | --- | --- | --- |
|  | **T2DM** | **Control** | **aSMD** | **T2DM** | **Control** | **aSMD** |
| **Patients (n)** | 967 | 11,896 |  | 633 | 633 |  |
| **Age group** |  |  |  |  |  |  |
| 15 - 19 | 0.4 | 1.6 | 0.12 | 0.6 | 0.3 | 0.05 |
| 20 - 24 | 0.3 | 3.6 | 0.24 | 0.5 | 0.9 | 0.06 |
| 25 - 29 | 1.0 | 3.3 | 0.15 | 1.3 | 1.6 | 0.03 |
| 30 - 34 | 1.8 | 4.8 | 0.17 | 1.7 | 2.1 | 0.02 |
| 35 - 39 | 3.9 | 7.6 | 0.16 | 3.9 | 3.0 | 0.05 |
| 40 - 44 | 5.8 | 9.1 | 0.12 | 6.3 | 6.2 | 0.01 |
| 45 - 49 | 8.7 | 10.0 | 0.05 | 9.5 | 9.0 | 0.02 |
| 50 - 54 | 14.1 | 10.5 | 0.11 | 14.4 | 15.2 | 0.02 |
| 55 - 59 | 13.7 | 11.2 | 0.08 | 14.2 | 15.3 | 0.03 |
| 60 - 64 | 15.4 | 10.5 | 0.15 | 14.5 | 14.2 | 0.01 |
| 65 - 69 | 11.7 | 9.5 | 0.07 | 10.3 | 11.4 | 0.04 |
| 70 - 74 | 11.5 | 7.6 | 0.13 | 11.4 | 9.6 | 0.06 |
| 75 - 79 | 8.4 | 5.8 | 0.10 | 7.7 | 7.0 | 0.03 |
| 80 - 84 | 2.8 | 3.3 | 0.03 | 3.2 | 2.7 | 0.03 |
| 85 - 89 | 0.6 | 1.4 | 0.08 | 0.5 | 0.8 | 0.04 |
| **Female** | 47.7 | 52.0 | 0.09 | 47.6 | 46.9 | 0.01 |
| **Medical history (general)** |  |  |  |  |  |  |
| Acute respiratory disease | 2.0 | 0.7 | 0.11 | 1.1 | 1.1 | 0.00 |
| Chronic liver disease | 3.0 | 0.4 | 0.20 | 2.2 | 2.1 | 0.01 |
| Chronic obstructive lung disease | 0.9 | 0.5 | 0.05 | 0.6 | 0.9 | 0.04 |
| Dementia | 0.9 | 0.6 | 0.04 | 1.1 | 0.8 | 0.03 |
| Depressive disorder | 2.1 | 1.1 | 0.08 | 1.4 | 2.4 | 0.07 |
| Gastroesophageal reflux disease | 3.6 | 2.8 | 0.04 | 3.5 | 4.9 | 0.07 |
| Gastrointestinal hemorrhage | 1.0 | 0.4 | 0.07 | 1.3 | 1.4 | 0.01 |
| Human immunodeficiency virus infection | 0.2 | 0.1 | 0.02 | 0.2 | 0.3 | 0.03 |
| Hyperlipidemia | 9.9 | 2.8 | 0.29 | 6.3 | 7.4 | 0.04 |
| Lesion of liver | 4.0 | 1.1 | 0.19 | 3.3 | 3.6 | 0.02 |
| Obesity | 1.8 | 0.4 | 0.13 | 1.7 | 1.1 | 0.05 |
| Osteoarthritis | 1.9 | 1.1 | 0.07 | 1.7 | 1.6 | 0.01 |
| Urinary tract infectious disease | 1.6 | 0.3 | 0.13 | 1.6 | 1.1 | 0.04 |
| Viral hepatitis C | 0.5 | 0.1 | 0.08 | 0.2 | 0.3 | 0.03 |
| **Medical history (cardiovascular disease)** |  |  |  |  |  |  |
| Cerebrovascular disease | 3.2 | 1.7 | 0.10 | 2.8 | 3.9 | 0.06 |
| Coronary arteriosclerosis | 12.4 | 1.7 | 0.43 | 8.5 | 10.7 | 0.08 |
| Heart disease | 20.8 | 4.5 | 0.51 | 15.6 | 18.3 | 0.07 |
| Peripheral vascular disease | 3.8 | 0.3 | 0.25 | 1.1 | 1.4 | 0.03 |
| Pulmonary embolism | 0.3 | 0.1 | 0.04 | 0.5 | 0.8 | 0.04 |
| **Medical history (neoplasm)** |  |  |  |  |  |  |
| Hematologic neoplasm | 2.5 | 0.9 | 0.12 | 2.2 | 3.5 | 0.08 |
| Malignant neoplastic disease | 9.5 | 5.3 | 0.16 | 8.5 | 10.7 | 0.08 |
| Malignant tumor of breast | 0.8 | 0.8 | 0.00 | 0.6 | 1.3 | 0.07 |
| Malignant tumor of colon | 0.4 | 0.3 | 0.01 | 0.3 | 0.3 | 0.00 |
| Malignant tumor of lung | 0.4 | 0.5 | 0.01 | 0.6 | 0.8 | 0.02 |
| Primary malignant neoplasm of prostate | 0.5 | 0.4 | 0.02 | 0.8 | 1.6 | 0.07 |

Data are presented as %, unless otherwise stated. *Abbreviations: PS: propensity score; aSMD: absolute standardized mean difference.*

**Table E1-2.** Comparative analysis of baseline characteristics and comorbidity profiles between the type 2 diabetes and control cohorts before/after the 1:4 propensity score matching at AUMC

| **Characteristics** | **Before PS matching** | | | **After PS matching** | | |
| --- | --- | --- | --- | --- | --- | --- |
|  | **T2DM** | **Control** | **aSMD** | **T2DM** | **Control** | **aSMD** |
| **Patients (n)** | 967 | 11,896 |  | 633 | 1,710 |  |
| **Age group** |  |  |  |  |  |  |
| 15 - 19 | 0.4 | 1.6 | 0.12 | 0.6 | 0.4 | 0.01 |
| 20 - 24 | 0.3 | 3.6 | 0.24 | 0.5 | 0.6 | 0.04 |
| 25 - 29 | 1.0 | 3.3 | 0.15 | 1.3 | 1.2 | 0.04 |
| 30 - 34 | 1.8 | 4.8 | 0.17 | 1.7 | 1.5 | 0.04 |
| 35 - 39 | 3.9 | 7.6 | 0.16 | 3.9 | 1.9 | 0.05 |
| 40 - 44 | 5.8 | 9.1 | 0.12 | 6.3 | 4.2 | 0.00 |
| 45 - 49 | 8.7 | 10.0 | 0.05 | 9.5 | 5.6 | 0.02 |
| 50 - 54 | 14.1 | 10.5 | 0.11 | 14.4 | 10.4 | 0.06 |
| 55 - 59 | 13.7 | 11.2 | 0.08 | 14.2 | 8.2 | 0.02 |
| 60 - 64 | 15.4 | 10.5 | 0.15 | 14.5 | 8.4 | 0.01 |
| 65 - 69 | 11.7 | 9.5 | 0.07 | 10.3 | 6.5 | 0.03 |
| 70 - 74 | 11.5 | 7.6 | 0.13 | 11.4 | 5.7 | 0.05 |
| 75 - 79 | 8.4 | 5.8 | 0.10 | 7.7 | 3.9 | 0.04 |
| 80 - 84 | 2.8 | 3.3 | 0.03 | 3.2 | 1.6 | 0.02 |
| 85 - 89 | 0.6 | 1.4 | 0.08 | 0.5 | 0.6 | 0.07 |
| **Female** | 47.7 | 52.0 | 0.09 | 47.6 | 28.1 | 0.05 |
| **Medical history (general)** |  |  |  |  |  |  |
| Acute respiratory disease | 2.0 | 0.7 | 0.11 | 1.1 | 0.7 | 0.00 |
| Chronic liver disease | 3.0 | 0.4 | 0.20 | 2.2 | 1.0 | 0.02 |
| Chronic obstructive lung disease | 0.9 | 0.5 | 0.05 | 0.6 | 0.6 | 0.07 |
| Dementia | 0.9 | 0.6 | 0.04 | 1.1 | 0.6 | 0.02 |
| Depressive disorder | 2.1 | 1.1 | 0.08 | 1.4 | 1.5 | 0.06 |
| Gastroesophageal reflux disease | 3.6 | 2.8 | 0.04 | 3.5 | 2.4 | 0.04 |
| Gastrointestinal hemorrhage | 1.0 | 0.4 | 0.07 | 1.3 | 0.8 | 0.01 |
| Human immunodeficiency virus infection | 0.2 | 0.1 | 0.02 | 0.2 | 0.2 | 0.02 |
| Hyperlipidemia | 9.9 | 2.8 | 0.29 | 6.3 | 3.5 | 0.00 |
| Lesion of liver | 4.0 | 1.1 | 0.19 | 3.3 | 1.8 | 0.01 |
| Obesity | 1.8 | 0.4 | 0.13 | 1.7 | 0.6 | 0.06 |
| Osteoarthritis | 1.9 | 1.1 | 0.07 | 1.7 | 0.9 | 0.03 |
| Urinary tract infectious disease | 1.6 | 0.3 | 0.13 | 1.6 | 0.5 | 0.06 |
| Viral hepatitis C | 0.5 | 0.1 | 0.08 | 0.2 | 0.2 | 0.03 |
| **Medical history (cardiovascular disease)** |  |  |  |  |  |  |
| Cerebrovascular disease | 3.2 | 1.7 | 0.10 | 2.8 | 2.3 | 0.07 |
| Coronary arteriosclerosis | 12.4 | 1.7 | 0.43 | 8.5 | 5.0 | 0.07 |
| Heart disease | 20.8 | 4.5 | 0.51 | 15.6 | 8.8 | 0.06 |
| Peripheral vascular disease | 3.8 | 0.3 | 0.25 | 1.1 | 0.8 | 0.03 |
| Pulmonary embolism | 0.3 | 0.1 | 0.04 | 0.5 | 0.4 | 0.04 |
| **Medical history (neoplasm)** |  |  |  |  |  |  |
| Hematologic neoplasm | 2.5 | 0.9 | 0.12 | 2.2 | 1.7 | 0.07 |
| Malignant neoplastic disease | 9.5 | 5.3 | 0.16 | 8.5 | 6.0 | 0.09 |
| Malignant tumor of breast | 0.8 | 0.8 | 0.00 | 0.6 | 0.6 | 0.05 |
| Malignant tumor of colon | 0.4 | 0.3 | 0.01 | 0.3 | 0.3 | 0.04 |
| Malignant tumor of lung | 0.4 | 0.5 | 0.01 | 0.6 | 0.6 | 0.05 |
| Primary malignant neoplasm of prostate | 0.5 | 0.4 | 0.02 | 0.8 | 0.6 | 0.07 |

Data are presented as %, unless otherwise stated. *Abbreviations: PS: propensity score; aSMD: absolute standardized mean difference.***Table E1-3.** Comparative analysis of baseline characteristics and comorbidity profiles between the hypertension and control cohorts before/after the 1:1 propensity score matching at AUMC

| **Characteristics** | **Before PS matching** | | | **After PS matching** | | |
| --- | --- | --- | --- | --- | --- | --- |
|  | **HTN** | **Control** | **aSMD** | **HTN** | **Control** | **aSMD** |
| **Patients (n)** | 1,369 | 2,732 |  | 1,277 | 1,277 |  |
| **Age group** |  |  |  |  |  |  |
| 15 - 19 | 0.3 | 1.9 | 0.15 | <0.7 | 1.1 | 0.06 |
| 20 - 24 | 0.5 | 4.4 | 0.26 | <0.7 | 0.7 | 0.02 |
| 25 - 29 | 1.3 | 4.2 | 0.18 | 1.8 | 1.6 | 0.02 |
| 30 - 34 | 1.5 | 6 | 0.24 | 2.4 | 1.6 | 0.06 |
| 35 - 39 | 3.6 | 9.1 | 0.23 | 5.9 | 5.4 | 0.02 |
| 40 - 44 | 6.2 | 10.7 | 0.16 | 8.3 | 7.4 | 0.03 |
| 45 - 49 | 6.9 | 11.1 | 0.15 | 8.9 | 9.3 | 0.01 |
| 50 - 54 | 12.1 | 11.1 | 0.03 | 12.6 | 13.8 | 0.04 |
| 55 - 59 | 14.3 | 10.6 | 0.11 | 15.4 | 13.9 | 0.04 |
| 60 - 64 | 14.6 | 9.6 | 0.15 | 13.6 | 15.3 | 0.05 |
| 65 - 69 | 14 | 8 | 0.19 | 11.1 | 10.8 | 0.01 |
| 70 - 74 | 10.9 | 5.7 | 0.19 | 7.8 | 7.6 | 0.00 |
| 75 - 79 | 8 | 4 | 0.17 | 6.4 | 7.2 | 0.03 |
| 80 - 84 | 3.9 | 2.4 | 0.09 | 3.3 | 3.4 | 0.01 |
| 85 - 89 | 1.5 | 1 | 0.05 | 1.2 | 0.8 | 0.04 |
| 90 - 94 | 0.4 | 0.2 | 0.03 | <0.7 | <0.7 | 0.03 |
| **Female** | 47.6 | 51.3 | 0.07 | 52 | 50.3 | 0.03 |
| **Medical history (general)** |  |  |  |  |  |  |
| Acute respiratory disease | 1.8 | 0.4 | 0.13 | 1.3 | 0.7 | 0.07 |
| Chronic liver disease | 1.4 | 0.3 | 0.12 | 0.7 | <0.7 | 0.04 |
| Chronic obstructive lung disease | 1.5 | 0.1 | 0.15 | 0.9 | 0.7 | 0.03 |
| Dementia | 1 | 0.3 | 0.08 | 1.1 | 1.4 | 0.04 |
| Depressive disorder | 1.2 | 0.5 | 0.08 | 0.9 | 1.8 | 0.08 |
| Gastroesophageal reflux disease | 2.9 | 1.6 | 0.09 | 2.6 | 3.8 | 0.07 |
| Gastrointestinal hemorrhage | 1.3 | 0.2 | 0.13 | 0.7 | <0.7 | 0.04 |
| Human immunodeficiency virus infection | <0.3 | 0.1 | 0.01 | <0.7 | <0.7 | 0.03 |
| Hyperlipidemia | 8.3 | 1.8 | 0.30 | 6.3 | 6.4 | 0.00 |
| Lesion of liver | 2.7 | 0.5 | 0.17 | 1.6 | 1.2 | 0.03 |
| Obesity | 1.5 | 0.2 | 0.13 | 1.1 | 0.8 | 0.03 |
| Osteoarthritis | 1.7 | 0.6 | 0.10 | 1.6 | 1.8 | 0.02 |
| Urinary tract infectious disease | 0.8 | 0.1 | 0.10 | <0.7 | <0.7 | 0.03 |
| Viral hepatitis C | 0.5 | 0.1 | 0.07 | <0.7 | <0.7 | 0.03 |
| **Medical history (cardiovascular disease)** |  |  |  |  |  |  |
| Cerebrovascular disease | 3.9 | 0.9 | 0.20 | 2.2 | 3.7 | 0.09 |
| Coronary arteriosclerosis | 10.5 | 0.2 | 0.47 | 1.7 | 1.6 | 0.01 |
| Heart disease | 19.3 | 0.5 | 0.66 | 4.5 | 3.9 | 0.03 |
| Peripheral vascular disease | 2.4 | 0.2 | 0.20 | 1.3 | 1.2 | 0.01 |
| Pulmonary embolism | 0.6 | <0.1 | 0.10 | 0.7 | <0.7 | 0.06 |
| **Medical history (neoplasm)** |  |  |  |  |  |  |
| Hematologic neoplasm | 3.2 | 0.2 | 0.23 | 1.3 | 0.9 | 0.04 |
| Malignant neoplastic disease | 11.4 | 2.4 | 0.36 | 6.6 | 7.5 | 0.04 |
| Malignant tumor of breast | 1.1 | 0.7 | 0.05 | 1.6 | 1.7 | 0.01 |
| Malignant tumor of colon | 0.6 | 0.1 | 0.09 | <0.7 | <0.7 | 0.02 |
| Malignant tumor of lung | 1 | 0.2 | 0.11 | 0.8 | 0.9 | 0.01 |
| Primary malignant neoplasm of prostate | 0.8 | 0.1 | 0.10 | <0.7 | 0.7 | 0.04 |

Data are presented as %, unless otherwise stated. *Abbreviations: PS: propensity score; aSMD: absolute standardized mean difference.*

**Table E1-4.** Comparative analysis of baseline characteristics and comorbidity profiles between the hypertension and control cohorts before/after the 1:2 propensity score matching at AUMC

| **Characteristics** | **Before PS matching** | | | **After PS matching** | | |
| --- | --- | --- | --- | --- | --- | --- |
|  | **HTN** | **Control** | **aSMD** | **HTN** | **Control** | **aSMD** |
| **Patients (n)** | 1,369 | 2,732 |  | 1,277 | 1,277 |  |
| **Age group** |  |  |  |  |  |  |
| 15 - 19 | 0.3 | 1.9 | 0.15 | <0.7 | 0.7 | 0.03 |
| 20 - 24 | 0.5 | 4.4 | 0.26 | <0.7 | 0.9 | 0.05 |
| 25 - 29 | 1.3 | 4.2 | 0.18 | 1.8 | 1.7 | 0.01 |
| 30 - 34 | 1.5 | 6 | 0.24 | 2.4 | 1.8 | 0.04 |
| 35 - 39 | 3.6 | 9.1 | 0.23 | 5.9 | 5.4 | 0.02 |
| 40 - 44 | 6.2 | 10.7 | 0.16 | 8.3 | 7.6 | 0.02 |
| 45 - 49 | 6.9 | 11.1 | 0.15 | 8.9 | 9.5 | 0.02 |
| 50 - 54 | 12.1 | 11.1 | 0.03 | 12.6 | 14.6 | 0.06 |
| 55 - 59 | 14.3 | 10.6 | 0.11 | 15.4 | 13.2 | 0.06 |
| 60 - 64 | 14.6 | 9.6 | 0.15 | 13.6 | 13.5 | 0.00 |
| 65 - 69 | 14 | 8 | 0.19 | 11.1 | 11 | 0.00 |
| 70 - 74 | 10.9 | 5.7 | 0.19 | 7.8 | 8.3 | 0.02 |
| 75 - 79 | 8 | 4 | 0.17 | 6.4 | 7.6 | 0.04 |
| 80 - 84 | 3.9 | 2.4 | 0.09 | 3.3 | 3 | 0.01 |
| 85 - 89 | 1.5 | 1 | 0.05 | 1.2 | 0.9 | 0.03 |
| 90 - 94 | 0.4 | 0.2 | 0.03 | <0.7 | <0.4 | 0.01 |
| **Female** | 47.6 | 51.3 | 0.07 | 52 | 50.5 | 0.03 |
| **Medical history (general)** |  |  |  |  |  |  |
| Acute respiratory disease | 1.8 | 0.4 | 0.13 | 1.3 | 0.9 | 0.04 |
| Chronic liver disease | 1.4 | 0.3 | 0.12 | 0.7 | 0.6 | 0.01 |
| Chronic obstructive lung disease | 1.5 | 0.1 | 0.15 | 0.9 | 0.6 | 0.04 |
| Dementia | 1 | 0.3 | 0.08 | 1.1 | 1.3 | 0.02 |
| Depressive disorder | 1.2 | 0.5 | 0.08 | 0.9 | 1.6 | 0.06 |
| Gastroesophageal reflux disease | 2.9 | 1.6 | 0.09 | 2.6 | 3.4 | 0.04 |
| Gastrointestinal hemorrhage | 1.3 | 0.2 | 0.13 | 0.7 | <0.4 | 0.04 |
| Human immunodeficiency virus infection | <0.3 | 0.1 | 0.01 | <0.7 | <0.4 | 0.02 |
| Lesion of liver | 2.7 | 0.5 | 0.17 | 1.6 | 1.4 | 0.01 |
| Obesity | 1.5 | 0.2 | 0.13 | 1.1 | 0.9 | 0.01 |
| Osteoarthritis | 1.7 | 0.6 | 0.10 | 1.6 | 1.8 | 0.02 |
| Renal impairment | 4.6 | 0.1 | 0.29 | 0.8 | 0.9 | 0.01 |
| Rheumatoid arthritis | 1.1 | 1.5 | 0.03 | 1.8 | 2.1 | 0.02 |
| Urinary tract infectious disease | 0.8 | 0.1 | 0.10 | <0.7 | <0.4 | 0.01 |
| **Medical history (cardiovascular disease)** |  |  |  |  |  |  |
| Cerebrovascular disease | 3.9 | 0.9 | 0.20 | 2.2 | 3.6 | 0.08 |
| Coronary arteriosclerosis | 10.5 | 0.2 | 0.47 | 1.7 | 1.6 | 0.01 |
| Heart disease | 19.3 | 0.5 | 0.66 | 4.5 | 4.1 | 0.02 |
| Ischemic heart disease | 10.6 | 0.1 | 0.48 | 1.2 | 1.3 | 0.01 |
| Peripheral vascular disease | 2.4 | 0.2 | 0.20 | 1.3 | 0.9 | 0.04 |
| Pulmonary embolism | 0.6 | <0.1 | 0.10 | 0.7 | <0.4 | 0.05 |
| Venous thrombosis | 0.6 | <0.1 | 0.09 | <0.7 | <0.4 | 0.01 |
| **Medical history (neoplasm)** |  |  |  |  |  |  |
| Hematologic neoplasm | 3.2 | 0.2 | 0.23 | 1.3 | 0.9 | 0.04 |
| Malignant neoplasm of anorectum | 0.3 | <0.1 | 0.07 | <0.7 | <0.4 | 0.02 |
| Malignant neoplastic disease | 11.4 | 2.4 | 0.36 | 6.6 | 7.8 | 0.05 |
| Malignant tumor of breast | 1.1 | 0.7 | 0.05 | 1.6 | 2 | 0.03 |
| Malignant tumor of colon | 0.6 | 0.1 | 0.09 | <0.7 | <0.4 | 0.04 |
| Primary malignant neoplasm of prostate | 0.8 | 0.1 | 0.10 | <0.7 | 0.7 | 0.04 |

Data are presented as %, unless otherwise stated. *Abbreviations: PS: propensity score; aSMD: absolute standardized mean difference.*

**Table E1-5.** Comparative analysis of baseline characteristics and comorbidity profiles between the hypertension and control cohorts before/after the 1:4 propensity score matching at AUMC

| **Characteristics** | **Before PS matching** | | | **After PS matching** | | |
| --- | --- | --- | --- | --- | --- | --- |
|  | **HTN** | **Control** | **aSMD** | **HTN** | **Control** | **aSMD** |
| **Patients (n)** | 1,369 | 2,732 |  | 1,277 | 1,277 |  |
| **Age group** |  |  |  |  |  |  |
| 15 - 19 | 0.3 | 1.9 | 0.15 | <0.7 | 0.6 | 0.01 |
| 20 - 24 | 0.5 | 4.4 | 0.26 | <0.7 | 0.8 | 0.03 |
| 25 - 29 | 1.3 | 4.2 | 0.18 | 1.8 | 1.7 | 0.01 |
| 30 - 34 | 1.5 | 6 | 0.24 | 2.4 | 2.1 | 0.02 |
| 35 - 39 | 3.6 | 9.1 | 0.23 | 5.9 | 4.7 | 0.05 |
| 40 - 44 | 6.2 | 10.7 | 0.16 | 8.3 | 8.2 | 0.00 |
| 45 - 49 | 6.9 | 11.1 | 0.15 | 8.9 | 9.3 | 0.01 |
| 50 - 54 | 12.1 | 11.1 | 0.03 | 12.6 | 14 | 0.04 |
| 55 - 59 | 14.3 | 10.6 | 0.11 | 15.4 | 13.5 | 0.05 |
| 60 - 64 | 14.6 | 9.6 | 0.15 | 13.6 | 13.9 | 0.01 |
| 65 - 69 | 14 | 8 | 0.19 | 11.1 | 11.3 | 0.01 |
| 70 - 74 | 10.9 | 5.7 | 0.19 | 7.8 | 7.9 | 0.00 |
| 75 - 79 | 8 | 4 | 0.17 | 6.4 | 7.9 | 0.06 |
| 80 - 84 | 3.9 | 2.4 | 0.09 | 3.3 | 3 | 0.02 |
| 85 - 89 | 1.5 | 1 | 0.05 | 1.2 | 0.9 | 0.03 |
| 90 - 94 | 0.4 | 0.2 | 0.03 | <0.7 | <0.3 | 0.01 |
| **Female** | 47.6 | 51.3 | 0.07 | 52 | 50.1 | 0.04 |
| **Medical history (general)** |  |  |  |  |  |  |
| Acute respiratory disease | 1.8 | 0.4 | 0.13 | 1.3 | 0.9 | 0.04 |
| Chronic liver disease | 1.4 | 0.3 | 0.12 | 0.7 | 0.6 | 0.00 |
| Chronic obstructive lung disease | 1.5 | 0.1 | 0.15 | 0.9 | 0.6 | 0.04 |
| Dementia | 1 | 0.3 | 0.08 | 1.1 | 1.2 | 0.01 |
| Depressive disorder | 1.2 | 0.5 | 0.08 | 0.9 | 1.7 | 0.06 |
| Gastroesophageal reflux disease | 2.9 | 1.6 | 0.09 | 2.6 | 3.3 | 0.04 |
| Gastrointestinal hemorrhage | 1.3 | 0.2 | 0.13 | 0.7 | 0.5 | 0.03 |
| Human immunodeficiency virus infection | <0.3 | 0.1 | 0.01 | <0.7 | <0.3 | 0.02 |
| Hyperlipidemia | 8.3 | 1.8 | 0.30 | 6.3 | 6.4 | 0.00 |
| Lesion of liver | 2.7 | 0.5 | 0.17 | 1.6 | 1.4 | 0.01 |
| Obesity | 1.5 | 0.2 | 0.13 | 1.1 | 0.8 | 0.03 |
| Osteoarthritis | 1.7 | 0.6 | 0.10 | 1.6 | 1.9 | 0.03 |
| Pneumonia | 2.1 | 0.4 | 0.16 | 1.4 | 1.5 | 0.00 |
| Renal impairment | 4.6 | 0.1 | 0.29 | 0.8 | 0.9 | 0.01 |
| Rheumatoid arthritis | 1.1 | 1.5 | 0.03 | 1.8 | 2.4 | 0.04 |
| Ulcerative colitis | <0.3 | 0.1 | 0.02 | <0.7 | <0.3 | 0.02 |
| Urinary tract infectious disease | 0.8 | 0.1 | 0.10 | <0.7 | <0.3 | 0.01 |
| Viral hepatitis C | 0.5 | 0.1 | 0.07 | <0.7 | 0.3 | 0.04 |
| **Medical history (cardiovascular disease)** |  |  |  |  |  |  |
| Cerebrovascular disease | 3.9 | 0.9 | 0.20 | 2.2 | 3.6 | 0.08 |
| Coronary arteriosclerosis | 10.5 | 0.2 | 0.47 | 1.7 | 1.5 | 0.01 |
| Heart disease | 19.3 | 0.5 | 0.66 | 4.5 | 4.1 | 0.02 |
| Heart failure | 1.8 | <0.1 | 0.19 | <0.7 | <0.3 | 0.09 |
| Ischemic heart disease | 10.6 | 0.1 | 0.48 | 1.2 | 1.3 | 0.01 |
| Peripheral vascular disease | 2.4 | 0.2 | 0.20 | 1.3 | 0.9 | 0.04 |
| Pulmonary embolism | 0.6 | <0.1 | 0.10 | 0.7 | 0.3 | 0.05 |
| Venous thrombosis | 0.6 | <0.1 | 0.09 | <0.7 | 0.3 | 0.01 |
| **Medical history (neoplasm)** |  |  |  |  |  |  |
| Hematologic neoplasm | 3.2 | 0.2 | 0.23 | 1.3 | 0.9 | 0.04 |
| Malignant lymphoma | 0.5 | 0.1 | 0.08 | <0.7 | 0.3 | 0.01 |
| Malignant neoplasm of anorectum | 0.3 | <0.1 | 0.07 | <0.7 | <0.3 | 0.03 |
| Malignant neoplastic disease | 11.4 | 2.4 | 0.36 | 6.6 | 7.6 | 0.04 |
| Malignant tumor of breast | 1.1 | 0.7 | 0.05 | 1.6 | 1.9 | 0.02 |
| Malignant tumor of colon | 0.6 | 0.1 | 0.09 | <0.7 | <0.3 | 0.04 |
| Malignant tumor of lung | 1 | 0.2 | 0.11 | 0.8 | 0.7 | 0.01 |
| Primary malignant neoplasm of prostate | 0.8 | 0.1 | 0.10 | <0.7 | 0.7 | 0.04 |

Data are presented as %, unless otherwise stated. *Abbreviations: PS: propensity score; aSMD: absolute standardized mean difference.*

**Table E2-1.** Comparative analysis of baseline characteristics and comorbidity profiles between the type 2 diabetes and control cohorts before/after the 1:1 propensity score matching at SNUBH

| **Characteristics** | **Before PS matching** | | | **After PS matching** | | |
| --- | --- | --- | --- | --- | --- | --- |
|  | **T2DM** | **Control** | **aSMD** | **T2DM** | **Control** | **aSMD** |
| **Patients (n)** | 424 | 13,546 |  | 324 | 324 |  |
| **Age group** |  |  |  |  |  |  |
| 30 - 34 | 1.2 | 4 | 0.18 | 1.5 | <1.5 | 0.09 |
| 35 - 39 | 2.1 | 6.7 | 0.22 | 2.8 | 3.1 | 0.02 |
| 40 - 44 | 4.5 | 9.2 | 0.19 | 4.9 | 6.2 | 0.05 |
| 45 - 49 | 7.8 | 9.7 | 0.07 | 8 | 7.4 | 0.02 |
| 50 - 54 | 12.5 | 10.6 | 0.06 | 12.7 | 13.3 | 0.02 |
| 55 - 59 | 10.8 | 11.6 | 0.03 | 11.4 | 9.3 | 0.07 |
| 60 - 64 | 16.7 | 10.8 | 0.17 | 16.7 | 17.6 | 0.03 |
| 65 - 69 | 17.2 | 10.4 | 0.20 | 16.4 | 19.8 | 0.09 |
| 70 - 74 | 17.2 | 9.5 | 0.23 | 15.7 | 9.3 | 0.20 |
| 75 - 79 | 7.3 | 7.5 | 0.01 | 7.1 | 6.2 | 0.04 |
| 80 - 84 | 2.4 | 2.7 | 0.02 | 2.5 | 3.7 | 0.07 |
| **Medical history (general)** |  |  |  |  |  |  |
| Acute respiratory disease | 1.4 | 0.3 | 0.12 | 1.5 | <1.5 | 0.13 |
| Chronic liver disease | 1.4 | 1 | 0.04 | 1.5 | 2.5 | 0.07 |
| Chronic obstructive lung disease | <1.2 | 0.5 | 0.06 | <1.5 | 1.5 | 0.06 |
| Dementia | 1.2 | 0.8 | 0.04 | 1.5 | 2.2 | 0.05 |
| Depressive disorder | 1.4 | 0.9 | 0.05 | 1.5 | <1.5 | 0.06 |
| Gastroesophageal reflux disease | 2.1 | 1.9 | 0.01 | 2.5 | 3.7 | 0.07 |
| Gastrointestinal hemorrhage | <1.2 | 0.5 | 0.05 | <1.5 | 1.9 | 0.05 |
| Hyperlipidemia | 13.2 | 3.3 | 0.37 | 10.2 | 11.7 | 0.05 |
| Hypertensive disorder | 33.5 | 5.4 | 0.76 | 25.9 | 29 | 0.07 |
| Lesion of liver | 1.9 | 0.9 | 0.09 | 1.5 | 2.5 | 0.07 |
| Obesity | 1.7 | 0.5 | 0.11 | 1.9 | 2.2 | 0.02 |
| Osteoarthritis | 3.5 | 1.2 | 0.16 | 3.7 | 2.2 | 0.09 |
| Pneumonia | 2.1 | 0.8 | 0.11 | 1.5 | 2.2 | 0.05 |
| Renal impairment | 3.3 | 0.5 | 0.20 | 1.5 | 1.9 | 0.02 |
| Urinary tract infectious disease | 1.4 | 0.2 | 0.13 | <1.5 | <1.5 | 0.06 |
| Visual system disorder | 32.3 | 90.5 | 1.49 | 39.8 | 36.4 | 0.07 |
| **Medical history (cardiovascular disease)** |  |  |  |  |  |  |
| Atrial fibrillation | 2.4 | 0.9 | 0.11 | 2.5 | 2.8 | 0.02 |
| Cerebrovascular disease | 8.5 | 4.6 | 0.16 | 9 | 12.3 | 0.11 |
| Coronary arteriosclerosis | 6.6 | 1.2 | 0.28 | 5.9 | 6.2 | 0.01 |
| Heart disease | 19.1 | 4.9 | 0.45 | 16.7 | 15.4 | 0.03 |
| Heart failure | <1.2 | 0.1 | 0.11 | <1.5 | <1.5 | 0.04 |
| lschemic heart disease | 11.3 | 1.6 | 0.40 | 8.6 | 6.5 | 0.08 |
| Peripheral vascular disease | 1.4 | 0.1 | 0.16 | <1.5 | <1.5 | 0.04 |
| **Medical history (neoplasm)** |  |  |  |  |  |  |
| Hematologic neoplasm | <1.2 | 0.2 | 0.01 | <1.5 | <1.5 | 0.11 |
| Malignant lymphoma | <1.2 | 0.2 | 0.00 | <1.5 | <1.5 | 0.08 |
| Malignant neoplastic disease | 7.5 | 5 | 0.11 | 7.4 | 10.5 | 0.11 |
| Malignant tumor of colon | <1.2 | 0.4 | 0.02 | <1.5 | <1.5 | 0.08 |
| Malignant tumor of lung | <1.2 | 0.6 | 0.02 | <1.5 | <1.5 | 0.06 |
| Primary malignant neoplasm of prostate | <1.2 | 0.4 | 0.04 | <1.5 | 2.2 | 0.17 |

Data are presented as %, unless otherwise stated. *Abbreviations: PS: propensity score; aSMD: absolute standardized mean difference.````````````````````````````````````````***Table E2-2.** Comparative analysis of baseline characteristics and comorbidity profiles between the type 2 diabetes and control cohorts before/after the 1:2 propensity score matching at SNUBH

| **Characteristics** | **Before PS matching** | | | **After PS matching** | | |
| --- | --- | --- | --- | --- | --- | --- |
|  | **T2DM** | **Control** | **aSMD** | **T2DM** | **Control** | **aSMD** |
| **Patients (n)** | 424 | 13,546 |  | 324 | 558 |  |
| **Age group** |  |  |  |  |  |  |
| 30 - 34 | 1.2 | 4 | 0.18 | 1.5 | 1.2 | 0.03 |
| 35 - 39 | 2.1 | 6.7 | 0.22 | 2.8 | 2.9 | 0.01 |
| 40 - 44 | 4.5 | 9.2 | 0.19 | 4.9 | 5.2 | 0.01 |
| 45 - 49 | 7.8 | 9.7 | 0.07 | 8 | 8.3 | 0.01 |
| 50 - 54 | 12.5 | 10.6 | 0.06 | 12.7 | 12.3 | 0.01 |
| 55 - 59 | 10 8 | 11.6 | 0.03 | 11 4 | 9.9 | 0.05 |
| 60 - 64 | 16.7 | 10.8 | 0.17 | 16.7 | 16.8 | 0.00 |
| 65 - 69 | 17.2 | 10.4 | 0.20 | 16.4 | 19.6 | 0.08 |
| 70 - 74 | 17.2 | 9.5 | 0.23 | 15.7 | 9.4 | 0.19 |
| 80 - 84 | 2.4 | 2.7 | 0.02 | 2.5 | 4 | 0.09 |
| **Female** | 36.8 | 52.1 | 0.31 | 38.6 | 39.8 | 0.03 |
| **Medical history (general)** |  |  |  |  |  |  |
| Acute respiratory disease | 1.4 | 0.3 | 0.12 | 1.5 | <0.9 | 0.13 |
| Chronic liver disease | 1.4 | 1 | 0.04 | 1.5 | 2 | 0.04 |
| Chronic obstructive lung disease | <1.2 | 0.5 | 0.06 | <1.5 | 1.5 | 0.06 |
| Dementia | 1.2 | 0.8 | 0.04 | 1.5 | 1.9 | 0.02 |
| Depressive disorder | 1.4 | 0.9 | 0 05 | 1.5 | 1.1 | 0.04 |
| Gastroesophageal reflux disease | 2.1 | 1.9 | 0 01 | 2.5 | 3.5 | 0.06 |
| Gastrointestinal hemorrhage | <1.2 | 0.5 | 0 05 | <1.5 | 1.7 | 0.04 |
| Hyperlipidemia | 13 2 | 3.3 | 0 37 | 10.2 | 12.8 | 0.08 |
| Hyperlipidemia |  | 1.8 | 0.30 | 6.3 | 6.4 | 0.00 |
| Hypertensive disorder | 33.5 | 5.4 | 0 76 | 25.9 | 30.4 | 0.10 |
| Lesion of liver | 1.9 | 0.9 | 0.09 | 1.5 | 2.6 | 0.08 |
| Obesity | 1.7 | 0.5 | 0.11 | 1.9 | 1.7 | 0.01 |
| Osteoarthritis | 3.5 | 1.2 | 0.16 | 3.7 | 2.5 | 0.07 |
| Pneumonia | 2.1 | 0.8 | 0.11 | 1.5 | 1.9 | 0.02 |
| Urinary tract infectious disease | 1.4 | 0.2 | 0.13 | <1.5 | <0.9 | 0.06 |
| Visual system disorder | 32.3 | 90.5 | 1.49 | 39.8 | 37.2 | 0.05 |
| **Medical history (cardiovascular disease)** |  |  |  |  |  |  |
| Atrial fibrillation | 2.4 | 0 9 | 0.11 | 2.5 | 3 1 | 0.04 |
| Cerebrovascular disease | 8.5 | 4.6 | 0.16 | 9 | 11.7 | 0.09 |
| Coronary arteriosclerosis | 6.6 | 1.2 | 0.28 | 5.9 | 5.7 | 0.01 |
| Heart disease | 19.1 | 4.9 | 0.45 | 16.7 | 17.3 | 0.02 |
| Heart failure | <1.2 | 0.1 | 0.11 | <1.5 | <0.9 | 0.02 |
| lschemic heart disease | 11 3 | 1.6 | 0.40 | 8.6 | 8.2 | 0.02 |
| Peripheral vascular disease | 1.4 | 0.1 | 0.16 | <1.5 | <0.9 | 0 04 |
| **Medical history (neoplasm)** |  |  |  |  |  |  |
| Hematologic neoplasm | <1.2 | 0.2 | 0 01 | <1.5 | <0.9 | 0 06 |
| Malignant lymphoma | <1.2 | 0.2 | 0.00 | <1.5 | <0.9 | 0.04 |
| Malignant neoplastic disease | 7.5 | 5 | 0 11 | 7.4 | 10.2 | 0.10 |
| Malignant tumor of colon | <1.2 | 0.4 | 0 02 | <1.5 | <0.9 | 0.06 |
| Malignant tumor of lung | <1.2 | 0.6 | 0 02 | <1.5 | 1.1 | 0.05 |
| Primary malignant neoplasm of prostate | <1.2 | 0.4 | 0 04 | <1.5 | 1.5 | 0.13 |

Data are presented as %, unless otherwise stated. *Abbreviations: PS: propensity score; aSMD: absolute standardized mean difference.***Table E2-3.** Comparative analysis of baseline characteristics and comorbidity profiles between the type 2 diabetes and control cohorts before/after the 1:4 propensity score matching at SNUBH

| **Characteristics** | **Before PS matching** | | | **After PS matching** | | |
| --- | --- | --- | --- | --- | --- | --- |
|  | **T2DM** | **Control** | **aSMD** | **T2DM** | **Control** | **aSMD** |
| **Patients (n)** | 424 | 13.546 |  | 324 | 973 |  |
| **Age group** |  |  |  |  |  |  |
| 20-24 | <1.2 | 2.5 | 0.20 | <1.5 | 0.5 | 0.04 |
| 30-34 | 1.2 | 4 | 0.18 | 1.5 | 1.5 | 0.01 |
| 35-39 | 2.1 | 6.7 | 0.22 | 2.8 | 2.7 | 0.01 |
| 40-44 | 4.5 | 9.2 | 0.19 | 4.9 | 4.8 | 0.01 |
| 45-49 | 7.8 | 9.7 | 0.07 | 8 | 8.8 | 0.03 |
| 50-54 | 12.5 | 10.6 | 0.06 | 12.7 | 12 | 0.02 |
| 55-59 | 10.8 | 11.6 | 0.03 | 11.4 | 9.3 | 0.07 |
| 60-64 | 16.7 | 10.8 | 0.17 | 16.7 | 17.3 | 0.02 |
| 65-69 | 17.2 | 10.4 | 0.20 | 16.4 | 19 | 0.07 |
| 70-74 | 17.2 | 9.5 | 0.23 | 15.7 | 11.1 | 0.14 |
| 75-79 | 7.3 | 7.5 | 0.01 | 7.1 | 6.6 | 0.02 |
| 80-84 | 2.4 | 2.7 | 0.02 | 2.5 | 3.5 | 0.06 |
| **Female** | 36.8 | 52.1 | 0.31 | 38.6 | 38.4 | 0.00 |
| **Medical history (general)** |  |  |  |  |  |  |
| Acute respiratory disease | 1.4 | 0.3 | 0.12 | 1.5 | <0.5 | 0.13 |
| Chronic liver disease | 1.4 | 1 | 0.04 | 1.5 | 1.8 | 0.02 |
| Chronic obstructive lung disease | <1.2 | 0.5 | 0.06 | <1.5 | 1.6 | 0.06 |
| Dementia | 1.2 | 0.8 | 0.04 | 1.5 | 2.2 | 0.05 |
| Depressive disorder | 1.4 | 0.9 | 0.05 | 1.5 | 1 | 0.05 |
| Gastroesophageal reflux disease | 2.1 | 1.9 | 0.01 | 2.5 | 3.5 | 0.06 |
| Gastrointestinal hemorrhage | <1.2 | 0.5 | 0.05 | <1.5 | 1.1 | 0.01 |
| Hyperlipidemia | 13.2 | 3.3 | 0.37 | 10.2 | 13.3 | 0.10 |
| Hypertensive disorder | 33.5 | 5.4 | 0.76 | 25.9 | 29.8 | 0.09 |
| Lesion of liver | 1.9 | 0.9 | 0.09 | 1.5 | 2.4 | 0.06 |
| Obesity | 1.7 | 0.5 | 0.11 | 1.9 | 1.7 | 0.01 |
| Osteoarthritis | 3.5 | 1.2 | 0.16 | 3.7 | 2 | 0.10 |
| Pneumonia | 2.1 | 0.8 | 0.11 | 1.5 | 1.8 | 0.02 |
| Renal impairment | 3.3 | 0.5 | 0.20 | 1.5 | 1.6 | 0.00 |
| Urinary tract infectious disease | 1.4 | 0.2 | 0.13 | <1.5 | <0.5 | 0.09 |
| Visual system disorder | 32.3 | 90.5 | 1.49 | 39.8 | 37.1 | 0.06 |
| **Medical history (cardiovascular disease)** |  |  |  |  |  |  |
| Atrial fibrillation | 2.4 | 0.9 | 0.11 | 2.5 | 3 | 0.03 |
| Cerebrovascular disease | 8.5 | 4.6 | 0.16 | 9 | 11.6 | 0.09 |
| Coronary arteriosclerosis | 6.6 | 1.2 | 0.28 | 5.9 | 5.6 | 0.01 |
| Heart disease | 19.1 | 4.9 | 0.45 | 16.7 | 16.4 | 0.01 |
| Ischemic heart disease | 11.3 | 1.6 | 0.40 | 8.6 | 8 | 0.02 |
| Peripheral vascular disease | 1.4 | 0.1 | 0.16 | <1.5 | <0.5 | 0.03 |
| Venous thrombosis | <1.2 | 0.1 | 0.04 | <1.5 | <0.5 | 0.01 |
| **Medical history (neoplasm)** |  |  |  |  |  |  |
| Hematologic neoplasm | <1.2 | 0.2 | 0.01 | <1.5 | 0.8 | 0.06 |
| Malignant neoplastic disease | 7.5 | 5 | 0.11 | 7.4 | 9.2 | 0.06 |
| Malignant tumor of colon | <1.2 | 0.4 | 0.02 | <1.5 | 0.9 | 0.07 |
| Malignant tumor of lung | <1.2 | 0.6 | 0.02 | <1.5 | 1 | 0.04 |
| Malignant tumor of urinary bladder | <1.2 | 0.1 | 0.11 | <1.5 | <0.5 | 0.12 |
| Primary malignant neoplasm of prostate | <1.2 | 0.4 | 0.04 | <1.5 | 1.3 | 0.11 |

Data are presented as %, unless otherwise stated. *Abbreviations: PS: propensity score; aSMD: absolute standardized mean difference.***Table E2-4.** Comparative analysis of baseline characteristics and comorbidity profiles between the hypertension and control cohorts before/after the 1:1 propensity score matching at SNUBH

| **Characteristics** | **Before PS matching** | | | **After PS matching** | | |
| --- | --- | --- | --- | --- | --- | --- |
|  | **HTN** | **Control** | **aSMD** | **HTN** | **Control** | **aSMD** |
| **Patients (n)** | 1,171 | 9,957 |  | 641 | 641 |  |
| **Age group** |  |  |  |  |  |  |
| 25-29 | <0.4 | 3.4 | 0.24 | <0.8 | 0.8 | 0.04 |
| 30-34 | 0.7 | 4.8 | 0.26 | 1.2 | 1.9 | 0.05 |
| 35-39 | 2.3 | 8.1 | 0.26 | 3.6 | 4.1 | 0.02 |
| 40-44 | 4.8 | 10.5 | 0.22 | 6.7 | 6.6 | 0.01 |
| 45-49 | 7.6 | 10.5 | 0.10 | 8.9 | 8.6 | 0.01 |
| 50-54 | 11.3 | 11 | 0.01 | 12.6 | 12.2 | 0.01 |
| 55-59 | 12 | 11.8 | 0.00 | 12 | 14.5 | 0.07 |
| 60-64 | 16.6 | 10.5 | 0.18 | 16.8 | 14.7 | 0.06 |
| 65-69 | 17.4 | 8.8 | 0.26 | 15 | 16.8 | 0.05 |
| 70-74 | 16.1 | 7.6 | 0.27 | 13.3 | 10.1 | 0.10 |
| 75-79 | 8.5 | 5.7 | 0.11 | 6.6 | 6.2 | 0.01 |
| **Female** | 49.4 | 52.5 | 0.06 | 51 | 48 | 0.06 |
| **Medical history (general)** |  |  |  |  |  |  |
| Acute respiratory disease | 0.6 | 0.3 | 0.04 | <0.8 | 0.8 | 0.02 |
| Chronic liver disease | 1.8 | 0.7 | 0.10 | 1.7 | 2.2 | 0.03 |
| Dementia | 0.7 | 0.5 | 0.03 | <0.8 | 1.1 | 0.09 |
| Depressive disorder | 1.7 | 0.6 | 0.11 | 1.4 | 0.9 | 0.04 |
| Diabetes mellitus | 19.6 | 2.4 | 0.57 | 12.3 | 14.8 | 0.07 |
| Gastroesophageal reflux disease | 3 | 1.3 | 0.12 | 2.2 | 3.1 | 0.06 |
| Gastrointestinal hemorrhage | 1.2 | 0.4 | 0.10 | 0.8 | 1.1 | 0.03 |
| Hyperlipidemia | 16.3 | 1.6 | 0.53 | 6.9 | 9.7 | 0.10 |
| Lesion of liver | 1.3 | 0.6 | 0.07 | 0.9 | 1.1 | 0.02 |
| Obesity | 1.9 | 0.4 | 0.14 | 1.6 | 1.7 | 0.01 |
| Osteoarthritis | 2.7 | 0.8 | 0.14 | 2 | 2.3 | 0.02 |
| Pneumonia | 1.8 | 0.4 | 0.14 | 1.4 | 1.2 | 0.01 |
| Psoriasis | 0.5 | 0.1 | 0.08 | <0.8 | <0.8 | 0.06 |
| Rheumatoid arthritis | 0.9 | 0.2 | 0.09 | 0.8 | <0.8 | 0.04 |
| Urinary tract infectious disease | 0.6 | 0.2 | 0.07 | <0.8 | <0.8 | 0.03 |
| Viral hepatitis C | 0.4 | 0.1 | 0.07 | <0.8 | <0.8 | 0.03 |
| **Medical history (cardiovascular disease)** |  |  |  |  |  |  |
| Atrial fibrillation | 4.1 | 0.1 | 0.28 | 1.1 | <0.8 | 0.05 |
| Cerebrovascular disease | 15.3 | 2 | 0.49 | 7.2 | 7.6 | 0.02 |
| Coronary arteriosclerosis | 5.6 | 0.3 | 0.32 | 1.9 | 2.2 | 0.02 |
| Heart disease | 22.5 | 0.8 | 0.72 | 6.4 | 6.7 | 0.01 |
| Ischemic heart disease | 10.4 | 0.2 | 0.47 | 1.7 | 2 | 0.02 |
| **Medical history (neoplasm)** |  |  |  |  |  |  |
| Malignant lymphoma | <0.4 | 0.1 | 0.04 | <0.8 | <0.8 | 0.03 |
| Malignant neoplastic disease | 6.4 | 3.3 | 0.14 | 5.1 | 6.6 | 0.06 |
| Malignant tumor of breast | 0.5 | 0.3 | 0.03 | <0.8 | 0.8 | 0.04 |
| Malignant tumor of colon | 0.6 | 0.2 | 0.07 | <0.8 | <0.8 | 0.03 |
| Malignant tumor of lung | 0.4 | 0.3 | 0.03 | <0.8 | <0.8 | 0.03 |
| Malignant tumor of urinary bladder | <0.4 | <0.1 | 0.06 | <0.8 | <0.8 | 0.03 |
| Primary malignant neoplasm of prostate | 0.6 | 0.3 | 0.05 | <0.8 | <0.8 | 0.03 |

Data are presented as %, unless otherwise stated. *Abbreviations: PS: propensity score; aSMD: absolute standardized mean difference.***Table E2-5.** Comparative analysis of baseline characteristics and comorbidity profiles between the hypertension and control cohorts before/after the 1:2 propensity score matching at SNUBH

| **Characteristics** | **Before PS matching** | | | **After PS matching** | | |
| --- | --- | --- | --- | --- | --- | --- |
|  | **HTN** | **Control** | **aSMD** | **HTN** | **Control** | **aSMD** |
| **Patients (n)** | 1,171 | 9,957 |  | 641 | 1,021 |  |
| **Age group** |  |  |  |  |  |  |
| 20-24 | <0.4 | 3.1 | 0.23 | <0.8 | <0.5 | 0.01 |
| 25-29 | <0.4 | 3.4 | 0.24 | <0.8 | 0.9 | 0.05 |
| 30-34 | 0.7 | 4.8 | 0.26 | 1.2 | 1.9 | 0.05 |
| 40-44 | 4.8 | 10.5 | 0.22 | 6.7 | 6.3 | 0.02 |
| 45-49 | 7.6 | 10.5 | 0.10 | 8.9 | 9.4 | 0.02 |
| 50-54 | 11.3 | 11 | 0.01 | 12.6 | 10.9 | 0.05 |
| 55-59 | 12 | 11.8 | 0.00 | 12 | 13.1 | 0.03 |
| 60-64 | 16.6 | 10.5 | 0.18 | 16.8 | 14.6 | 0.06 |
| 65-69 | 17.4 | 8.8 | 0.26 | 15 | 15.8 | 0.02 |
| 70-74 | 16.1 | 7.6 | 0.27 | 13.3 | 12.6 | 0.02 |
| 75-79 | 8.5 | 5.7 | 0.11 | 6.6 | 8 | 0.05 |
| 80-84 | 2.1 | 1.8 | 0.02 | 2.3 | 2 | 0.02 |
| **Female** | 49.4 | 52.5 | 0.06 | 51 | 49.6 | 0.03 |
| **Medical history (general)** |  |  |  |  |  |  |
| Acute respiratory disease | 0.6 | 0.3 | 0.04 | <0.8 | 0.9 | 0.03 |
| Chronic liver disease | 1.8 | 0.7 | 0.10 | 1.7 | 2.3 | 0.04 |
| Dementia | 0.7 | 0.5 | 0.03 | <0.8 | 0.9 | 0.07 |
| Depressive disorder | 1.7 | 0.6 | 0.11 | 1.4 | 1.2 | 0.01 |
| Diabetes mellitus | 19.6 | 2.4 | 0.57 | 12.3 | 14.8 | 0.07 |
| Gastroesophageal reflux disease | 3 | 1.3 | 0.12 | 2.2 | 2.9 | 0.04 |
| Gastrointestinal hemorrhage | 1.2 | 0.4 | 0.10 | 0.8 | 1.1 | 0.03 |
| Hyperlipidemia | 16.3 | 1.6 | 0.53 | 6.9 | 9.4 | 0.09 |
| Lesion of liver | 1.3 | 0.6 | 0.07 | 0.9 | 1.3 | 0.04 |
| Obesity | 1.9 | 0.4 | 0.14 | 1.6 | 2 | 0.04 |
| Osteoarthritis | 2.7 | 0.8 | 0.14 | 2 | 2.4 | 0.03 |
| Pneumonia | 1.8 | 0.4 | 0.14 | 1.4 | 1.1 | 0.03 |
| **Medical history (cardiovascular disease)** |  |  |  |  |  |  |
| Atrial fibrillation | 4.1 | 0.1 | 0.28 | 1.1 | 0.7 | 0.04 |
| Cerebrovascular disease | 15.3 | 2 | 0.49 | 7.2 | 7.8 | 0.02 |
| Coronary arteriosclerosis | 5.6 | 0.3 | 0.32 | 1.9 | 2.2 | 0.02 |
| Heart disease | 22.5 | 0.8 | 0.72 | 6.4 | 7 | 0.03 |
| Ischemic heart disease | 10.4 | 0.2 | 0.47 | 1.7 | 2.2 | 0.03 |
| **Medical history (neoplasm)** |  |  |  |  |  |  |
| Hematologic neoplasm | <0.4 | <0.1 | 0.04 | <0.8 | <0.5 | 0.02 |
| Malignant lymphoma | <0.4 | 0.1 | 0.04 | <0.8 | <0.5 | 0.02 |
| Malignant neoplastic disease | 6.4 | 3.3 | 0.14 | 5.1 | 6.2 | 0.04 |
| Malignant tumor of breast | 0.5 | 0.3 | 0.03 | <0.8 | 0.6 | 0.02 |
| Malignant tumor of colon | 0.6 | 0.2 | 0.07 | <0.8 | <0.5 | 0.03 |
| Malignant tumor of lung | 0.4 | 0.3 | 0.03 | <0.8 | <0.5 | 0.04 |
| Malignant tumor of urinary bladder | <0.4 | <0.1 | 0.06 | <0.8 | <0.5 | 0.03 |
| Primary malignant neoplasm of prostate | 0.6 | 0.3 | 0.05 | <0.8 | <0.5 | 0.03 |

Data are presented as %, unless otherwise stated. *Abbreviations: PS: propensity score; aSMD: absolute standardized mean difference.*

**Table E2-6.** Comparative analysis of baseline characteristics and comorbidity profiles between the hypertension and control cohorts before/after the 1:4 propensity score matching at SNUBH

| **Characteristics** | **Before PS matching** | | | **After PS matching** | | |
| --- | --- | --- | --- | --- | --- | --- |
|  | **HTN** | **Control** | **aSMD** | **HTN** | **Control** | **aSMD** |
| **Patients (n)** | 1,171 | 9,957 |  | 641 | 1,551 |  |
| **Age group** |  |  |  |  |  |  |
| 20-24 | <0.4 | 3.1 | 0.23 | <0.8 | 0.4 | 0.01 |
| 25-29 | <0.4 | 3.4 | 0.24 | <0.8 | 0.9 | 0.05 |
| 30-34 | 0.7 | 4.8 | 0.26 | 1.2 | 1.9 | 0.05 |
| 35-39 | 2.3 | 8.1 | 0.26 | 3.6 | 3.1 | 0.03 |
| 40-44 | 4.8 | 10.5 | 0.22 | 6.7 | 5.8 | 0.04 |
| 45-49 | 7.6 | 10.5 | 0.10 | 8.9 | 9 | 0.00 |
| 50-54 | 11.3 | 11 | 0.01 | 12.6 | 11.4 | 0.04 |
| 55-59 | 12 | 11.8 | 0.00 | 12 | 12.4 | 0.01 |
| 60-64 | 16.6 | 10.5 | 0.18 | 16.8 | 15.5 | 0.04 |
| 65-69 | 17.4 | 8.8 | 0.26 | 15 | 15.8 | 0.02 |
| 70-74 | 16.1 | 7.6 | 0.27 | 13.3 | 12.9 | 0.01 |
| 75-79 | 8.5 | 5.7 | 0.11 | 6.6 | 8.7 | 0.08 |
| 80-84 | 2.1 | 1.8 | 0.02 | 2.3 | 1.8 | 0.04 |
| **Female** | 49.4 | 52.5 | 0.06 | 51 | 48.9 | 0.04 |
| **Medical history (general)** |  |  |  |  |  |  |
| Acute respiratory disease | 0.6 | 0.3 | 0.04 | <0.8 | 0.7 | 0.01 |
| Chronic liver disease | 1.8 | 0.7 | 0.10 | 1.7 | 2.1 | 0.03 |
| Dementia | 0.7 | 0.5 | 0.03 | <0.8 | 0.8 | 0.06 |
| Depressive disorder | 1.7 | 0.6 | 0.11 | 1.4 | 1.1 | 0.02 |
| Diabetes mellitus | 19.6 | 2.4 | 0.57 | 12.3 | 15.2 | 0.08 |
| Gastroesophageal reflux disease | 3 | 1.3 | 0.12 | 2.2 | 3.2 | 0.06 |
| Gastrointestinal hemorrhage | 1.2 | 0.4 | 0.10 | 0.8 | 1.1 | 0.03 |
| Hyperlipidemia | 16.3 | 1.6 | 0.53 | 6.9 | 9.5 | 0.10 |
| Lesion of liver | 1.3 | 0.6 | 0.07 | 0.9 | 1.3 | 0.04 |
| Obesity | 1.9 | 0.4 | 0.14 | 1.6 | 2 | 0.04 |
| Osteoarthritis | 2.7 | 0.8 | 0.14 | 2 | 2.2 | 0.01 |
| Pneumonia | 1.8 | 0.4 | 0.14 | 1.4 | 1.2 | 0.02 |
| Psoriasis | 0.5 | 0.1 | 0.08 | <0.8 | <0.3 | 0.06 |
| Renal impairment | 2.2 | 0.1 | 0.20 | 0.9 | 0.8 | 0.01 |
| Rheumatoid arthritis | 0.9 | 0.2 | 0.09 | 0.8 | 0.7 | 0.01 |
| Urinary tract infectious disease | 0.6 | 0.2 | 0.07 | <0.8 | 0.6 | 0.07 |
| Viral hepatitis C | 0.4 | 0.1 | 0.07 | <0.8 | 0.4 | 0.01 |
| **Medical history (cardiovascular disease)** |  |  |  |  |  |  |
| Atrial fibrillation | 4.1 | 0.1 | 0.28 | 1.1 | 0.8 | 0.03 |
| Cerebrovascular disease | 15.3 | 2 | 0.49 | 7.2 | 8 | 0.03 |
| Coronary arteriosclerosis | 5.6 | 0.3 | 0.32 | 1.9 | 2.4 | 0.04 |
| Heart disease | 22.5 | 0.8 | 0.72 | 6.4 | 7.3 | 0.04 |
| Heart failure | 0.5 | <0.1 | 0.10 | <0.8 | <0.3 | 0.04 |
| Ischemic heart disease | 10.4 | 0.2 | 0.47 | 1.7 | 2.1 | 0.03 |
| **Medical history (neoplasm)** |  |  |  |  |  |  |
| Hematologic neoplasm | <0.4 | <0.1 | 0.04 | <0.8 | <0.3 | 0.04 |
| Malignant lymphoma | <0.4 | 0.1 | 0.04 | <0.8 | <0.3 | 0.02 |
| Malignant neoplastic disease | 6.4 | 3.3 | 0.14 | 5.1 | 6.8 | 0.07 |
| Malignant tumor of breast | 0.5 | 0.3 | 0.03 | <0.8 | 0.7 | 0.03 |
| Malignant tumor of colon | 0.6 | 0.2 | 0.07 | <0.8 | <0.3 | 0.02 |
| Malignant tumor of lung | 0.4 | 0.3 | 0.03 | <0.8 | 0.5 | 0.06 |
| Malignant tumor of urinary bladder | <0.4 | <0.1 | 0.06 | <0.8 | <0.3 | 0.03 |

Data are presented as %, unless otherwise stated. *Abbreviations: PS: propensity score; aSMD: absolute standardized mean difference.*

**Table E3-1.** A mixed-effects regression analysis of OCT values (1:1, AUMC)

| **Fixed effect** | **Estimate, β (95% CI)** | ***P* value** |
| --- | --- | --- |
| T2DM and non-T2DM groups | | |
| CMT | | |
| Time | 0.31 (-0.17, 0.78) | 0.20 |
| T2DM groups | 2.02 (-4.03, 8.08) | 0.51 |
| Age | 0.06 (-0.17, 0.29) | 0.60 |
| Gender (male) | 13.41 (7.84, 18.97) | <0.01 |
| Time × T2DM groups | -0.68 (-1.29, -0.07) | 0.03 |
| RNFL | | |
| Time | -0.61 (-0.99, -0.24) | 0.001 |
| T2DM groups | -3.26 (-7.04, 0.51) | 0.09 |
| Age | -0.13 (-0.26, 0.01) | 0.06 |
| Gender (male) | -3.38 (-6.54, -0.22) | 0.04 |
| Time × T2DM groups | 0.02 (-0.49, 0.54) | 0.93 |
| HTN and non-HTN groups | | |
| CMT | | |
| Time | 0.78 (0.33, 1.24) | <0.01 |
| HTN groups | 5.15 (-0.55, 10.85) | 0.08 |
| Age | -0.16 (-0.34, 0.02) | 0.08 |
| Gender (male) | 9.47 (4.63, 14.32) | <0.01 |
| Time × HTN groups | -0.71 (-1.43, 0.01) | 0.05 |
| RNFL | | |
| Time | -0.36 (-0.66, -0.06) | 0.02 |
| HTN groups | 0.61 (-3.04, 4.25) | 0.74 |
| Age | -0.23 (-0.35, -0.11) | <0.01 |
| Gender (male) | -4.04 (-7.14, -0.93) | 0.01 |
| Time × HTN groups | 0.17 (-0.29, 0.63) | 0.47 |

*Abbreviations: LB, lower bound; UB, upper bound; OCT, optical coherence tomography; T2DM, type 2 diabetes mellitus; HTN: hypertension; CMT: central macular thickness; RNFL: retinal nerve fiber layer*

**Table E3-2.** Group effects on the OCT values at different time points (1:1, AUMC, T2DM vs control cohorts)

|  | **Time period** | **T2DM group** | | |  | **Non-T2DM group** | | | ***P* value** |
| --- | --- | --- | --- | --- | --- | --- | --- | --- | --- |
|  |  | **Expected value** | **LB** | **UB** |  | **Expected value** | **LB** | **UB** |  |
| **CMT** | 0 year | 251 | 247 | 256 |  | 249 | 245 | 253 | 0.51 |
|  | 5 years | 249 | 245 | 254 |  | 251 | 247 | 255 | 0.64 |
|  | 10 years | 248 | 243 | 252 |  | 252 | 247 | 258 | 0.18 |
|  | 15 years | 246 | 239 | 252 |  | 254 | 247 | 261 | 0.08 |
| **RNFL** | 0 year | 85.2 | 82.1 | 88.3 |  | 88.5 | 86.2 | 90.7 | 0.09 |
|  | 5 years | 82.3 | 79.9 | 84.6 |  | 85.4 | 83.0 | 87.8 | 0.07 |
|  | 10 years | 79.3 | 76.3 | 82.3 |  | 82.3 | 78.7 | 86.0 | 0.20 |
|  | 15 years | 76.4 | 72.0 | 80.8 |  | 79.3 | 73.9 | 84.6 | 0.40 |

Results are averaged over the levels of age and gender. *Abbreviations: LB, lower bound; UB, upper bound; OCT, optical coherence tomography; T2DM, type 2 diabetes mellitus; CMT: central macular thickness; RNFL: retinal nerve fiber layer*

**Table E3-3.** Group effects on the OCT values at different time points (1:1, AUMC, HTN vs control cohorts)

|  | **Time period** | **HTN group** | | |  | **Non-HTN group** | | | ***P* value** |
| --- | --- | --- | --- | --- | --- | --- | --- | --- | --- |
|  |  | **Expected value** | **LB** | **UB** |  | **Expected value** | **LB** | **UB** |  |
| **CMT** | 0 year | 253 | 248 | 258 |  | 248 | 245 | 251 | 0.07 |
|  | 5 years | 253 | 249 | 257 |  | 252 | 248 | 255 | 0.56 |
|  | 10 years | 254 | 248 | 259 |  | 256 | 251 | 260 | 0.59 |
|  | 15 years | 254 | 246 | 262 |  | 259 | 253 | 266 | 0.28 |
| **RNFL** | 0 year | 88.1 | 85.0 | 91.3 |  | 87.5 | 85.6 | 89.4 | 0.74 |
|  | 5 years | 87.2 | 84.5 | 89.9 |  | 85.7 | 83.6 | 87.9 | 0.41 |
|  | 10 years | 86.3 | 82.8 | 89.8 |  | 84.0 | 80.8 | 87.2 | 0.32 |
|  | 15 years | 85.4 | 80.4 | 90.3 |  | 82.2 | 77.7 | 86.7 | 0.33 |

Results are averaged over the levels of age and gender. *Abbreviations: LB, lower bound; UB, upper bound; OCT, optical coherence tomography; HTN, hypertension; CMT: central macular thickness; RNFL: retinal nerve fiber layer*

**Table E3-4.** A mixed-effects regression analysis of OCT values (1:2, AUMC)

| **Fixed effect** | **Estimate, β (95% CI)** | ***P* value** |
| --- | --- | --- |
| T2DM and non-T2DM groups | | |
| CMT | | |
| Time | 0.36 (-0.09, 0.80) | 0.12 |
| T2DM groups | 1.47 (-3.94, 6.88) | 0.59 |
| Age | 0.11 (-0.06, 0.28) | 0.19 |
| Gender (male) | 14.11 (9.77, 18.46) | <0.01 |
| Time × T2DM groups | -0.69 (-1.34, -0.03) | 0.04 |
| RNFL | | |
| Time | -0.49 (-0.77, -0.22) | <0.01 |
| T2DM groups | -2.22 (-5.58, 1.14) | 0.20 |
| Age | -0.13 (-0.23, -0.02) | 0.02 |
| Gender (male) | -3.68 (-6.25, -1.10) | 0.01 |
| Time × T2DM groups | -0.13 (-0.55, 0.30) | 0.56 |
| HTN and non-HTN groups | | |
| CMT | | |
| Time | 0.84 (0.42, 1.26) | <0.01 |
| HTN groups | 6.85 (1.42, 12.27) | 0.01 |
| Age | -0.13 (-0.27, 0.01) | 0.08 |
| Gender (male) | 10.66 (6.80, 14.53) | <0.01 |
| Time × HTN groups | -0.81 (-1.57, -0.06) | 0.04 |
| RNFL | | |
| Time | -0.39 (-0.65, -0.13) | <0.01 |
| HTN groups | 1.85 (-1.77, 5.48) | 0.32 |
| Age | -0.19 (-0.29, -0.10) | <0.01 |
| Gender (male) | -3.83 (-6.46, -1.19) | <0.01 |
| Time × HTN groups | 0.20 (-0.28, 0.68) | 0.42 |

*Abbreviations: LB, lower bound; UB, upper bound; OCT, optical coherence tomography; T2DM, type 2 diabetes mellitus; HTN: hypertension; CMT: central macular thickness; RNFL: retinal nerve fiber layer*

**Table E3-5.** Group effects on the OCT values at different time points (1:2, AUMC, T2DM vs control cohorts)

|  | **Time period** | **T2DM group** | | |  | **Non-T2DM group** | | | ***P* value** |
| --- | --- | --- | --- | --- | --- | --- | --- | --- | --- |
|  |  | **Expected value** | **LB** | **UB** |  | **Expected value** | **LB** | **UB** |  |
| **CMT** | 0 year | 251 | 246 | 255 |  | 249 | 247 | 252 | 0.59 |
|  | 5 years | 249 | 245 | 253 |  | 251 | 248 | 254 | 0.43 |
|  | 10 years | 248 | 243 | 252 |  | 253 | 248 | 257 | 0.09 |
|  | 15 years | 246 | 239 | 252 |  | 255 | 248 | 261 | 0.05 |
| **RNFL** | 0 year | 85.6 | 82.6 | 88.5 |  | 87.8 | 86.2 | 89.4 | 0.20 |
|  | 5 years | 82.5 | 80.1 | 84.8 |  | 85.3 | 83.5 | 87.1 | 0.05 |
|  | 10 years | 79.4 | 76.5 | 82.2 |  | 82.9 | 80.1 | 85.6 | 0.08 |
|  | 15 years | 76.3 | 72.1 | 80.4 |  | 80..4 | 76.5 | 84.3 | 0.14 |

Results are averaged over the levels of age and gender. *Abbreviations: LB, lower bound; UB, upper bound; OCT, optical coherence tomography; T2DM, type 2 diabetes mellitus; CMT: central macular thickness; RNFL: retinal nerve fiber layer*

**Table E3-6.** Group effects on the OCT values at different time points (1:2, AUMC, HTN vs control cohorts)

|  | **Time period** | **HTN group** | | |  | **Non-HTN group** | | | ***P* value** |
| --- | --- | --- | --- | --- | --- | --- | --- | --- | --- |
|  |  | **Expected value** | **LB** | **UB** |  | **Expected value** | **LB** | **UB** |  |
| **CMT** | 0 year | 253 | 248 | 258 |  | 246 | 244 | 249 | 0.01 |
|  | 5 years | 253 | 249 | 258 |  | 251 | 248 | 253 | 0.27 |
|  | 10 years | 253 | 248 | 259 |  | 255 | 251 | 259 | 0.72 |
|  | 15 years | 254 | 245 | 262 |  | 259 | 253 | 265 | 0.29 |
| **RNFL** | 0 year | 88.3 | 85.0 | 91.7 |  | 86.5 | 85.0 | 88.0 | 0.32 |
|  | 5 years | 87.4 | 84.6 | 90.2 |  | 84.5 | 82.8 | 86.3 | 0.09 |
|  | 10 years | 86.4 | 82.7 | 90.2 |  | 82.6 | 79.9 | 85.2 | 0.09 |
|  | 15 years | 85.5 | 80.0 | 90.9 |  | 80.6 | 76.8 | 84.4 | 0.14 |

Results are averaged over the levels of age and gender. *Abbreviations: LB, lower bound; UB, upper bound; OCT, optical coherence tomography; HTN, hypertension; CMT: central macular thickness; RNFL: retinal nerve fiber layer*

**Table E3-7.** A mixed-effects regression analysis of OCT values (1:4, AUMC)

| **Fixed effect** | **Estimate, β (95% CI)** | ***P* value** |
| --- | --- | --- |
| T2DM and non-T2DM groups | | |
| CMT | | |
| Time | 0.37 (0.04. 0.69) | 0.03 |
| T2DM groups | 1.76 (-3.46, 6.98) | 0.51 |
| Age | 0.05 (-0.08, 0.18) | 0.44 |
| Gender (male) | 13.38 (9.71, 17.04) | <0.01 |
| Time × T2DM groups | -0.65 (-1.25, -0.06) | 0.03 |
| RNFL | | |
| Time | -1.00 (-1.57, -0.43) | 0.001 |
| T2DM groups | -5.32 (-12.40, 1.77) | 0.14 |
| Age | -0.05 (-0.21, 0.10) | 0.51 |
| Gender (male) | -5.62 (-9.62, -1.62) | 0.006 |
| Time × T2DM groups | 0.59 (-0.42, 1.60) | 0.25 |
| HTN and non-HTN groups | | |
| CMT | | |
| Time | -0.58 (0.29, 0.86) | <0.01 |
| HTN groups | -6.58 (-1.36, 11.80) | 0.01 |
| Age | -0.07 (-0.18, 0.05) | 0.27 |
| Gender (male) | 10.68 (7.40, 13.96) | <0.01 |
| Time × HTN groups | -0.60 (-1.27, 0.07) | 0.08 |
| RNFL | | |
| Time | -0.31 (-0.49, -0.13) | <0.01 |
| HTN groups | 2.33 (-1.16, 5.82) | 0.19 |
| Age | -0.18 (-0.26, -0.10) | <0.01 |
| Gender (male) | -4.37 (-6.57, -2.18) | <0.01 |
| Time × HTN groups | 0.10 (-0.34, 0.54) | 0.65 |

*Abbreviations: LB, lower bound; UB, upper bound; OCT, optical coherence tomography; T2DM, type 2 diabetes mellitus; HTN: hypertension; CMT: central macular thickness; RNFL: retinal nerve fiber layer*

**Table E3-8.** Group effects on the OCT values at different time points (1:4, AUMC, T2DM vs control cohorts)

|  | **Time period** | **T2DM group** | | |  | **Non-T2DM group** | | | ***P* value** |
| --- | --- | --- | --- | --- | --- | --- | --- | --- | --- |
|  |  | **Expected value** | **LB** | **UB** |  | **Expected value** | **LB** | **UB** |  |
| **CMT** | 0 year | 251 | 246 | 255 |  | 249 | 247 | 251 | 0.51 |
|  | 5 years | 249 | 245 | 253 |  | 251 | 248 | 253 | 0.51 |
|  | 10 years | 248 | 243 | 253 |  | 253 | 249 | 256 | 0.10 |
|  | 15 years | 246 | 240 | 253 |  | 254 | 250 | 259 | 0.04 |
| **RNFL** | 0 year | 83.7 | 77.1 | 90.2 |  | 89.0 | 86.3 | 91.7 | 0.14 |
|  | 5 years | 81.6 | 77.1 | 86.2 |  | 84.0 | 81.4 | 86.6 | 0.37 |
|  | 10 years | 79.6 | 73.9 | 85.3 |  | 79.0 | 74.3 | 83.8 | 0.88 |
|  | 15 years | 77.6 | 68.6 | 86.6 |  | 74.1 | 66.6 | 81.5 | 0.55 |

Results are averaged over the levels of age and gender. *Abbreviations: LB, lower bound; UB, upper bound; OCT, optical coherence tomography; T2DM, type 2 diabetes mellitus; CMT: central macular thickness; RNFL: retinal nerve fiber layer*

**Table E3-9.** Group effects on the OCT values at different time points (1:4, AUMC, HTN vs control cohorts)

|  | **Time period** | **HTN group** | | |  | **Non-HTN group** | | | ***P* value** |
| --- | --- | --- | --- | --- | --- | --- | --- | --- | --- |
|  |  | **Expected value** | **LB** | **UB** |  | **Expected value** | **LB** | **UB** |  |
| **CMT** | 0 year | 253 | 248 | 258 |  | 247 | 245 | 249 | 0.01 |
|  | 5 years | 253 | 249 | 257 |  | 250 | 248 | 252 | 0.13 |
|  | 10 years | 253 | 248 | 259 |  | 253 | 250 | 255 | 0.86 |
|  | 15 years | 253 | 245 | 261 |  | 255 | 251 | 260 | 0.58 |
| **RNFL** | 0 year | 88.6 | 85.3 | 91.9 |  | 86.3 | 85.1 | 87.5 | 0.19 |
|  | 5 years | 87.6 | 84.7 | 90.4 |  | 84.7 | 83.4 | 86.1 | 0.07 |
|  | 10 years | 86.5 | 82.8 | 90.2 |  | 83.2 | 81.3 | 85.1 | 0.11 |
|  | 15 years | 85.5 | 80.2 | 90.8 |  | 81.6 | 79.0 | 84.3 | 0.19 |

Results are averaged over the levels of age and gender. *Abbreviations: LB, lower bound; UB, upper bound; OCT, optical coherence tomography; HTN, hypertension; CMT: central macular thickness; RNFL: retinal nerve fiber layer*

**Table E4-1.** A mixed-effects regression analysis of OCT values (1:1, SNUBH)

| **Fixed effect** | **Estimate, β (95% CI)** | ***P* value** |
| --- | --- | --- |
| T2DM and non-T2DM groups | | |
| CMT | | |
| Time | 1.86 (-1.95, 5.68) | 0.337 |
| T2DM groups | -4.30 (-19.82, 11.22) | 0.586 |
| Age | -0.09 (-0.56, 0.37) | 0.686 |
| Gender (male) | 9.30 (-3.74, 22.33) | 0.162 |
| Time × T2DM groups | -2.73 (-7.24, 1.78) | 0.235 |
| RNFL | | |
| Time | -0.27 (-0.48, -0.06) | 0.01 |
| T2DM groups | 0.41 (-2.82, 3.64) | 0.80 |
| Age | -0.20 (-0.32, -0.07) | <0.01 |
| Gender (male) | -1.78 (-5.05, 1.50) | 0.29 |
| Time × T2DM groups | -0.05 (-0.29, 0.20) | 0.71 |
| HTN and non-HTN groups | | |
| CMT | | |
| Time | -1.44 (-4.75, 1.87) | 0.40 |
| HTN groups | -2.75 (-14.38, 8.88) | 0.64 |
| Age | -0.07 (-0.39, 0.25) | 0.67 |
| Gender (male) | 9.51 (2.04, 16.99) | 0.01 |
| Time × HTN groups | 0.93 (-2.76, 4.61) | 0.62 |
| RNFL | | |
| Time | -0.77 (-1.25, -0.29) | <0.01 |
| HTN groups | -4.13 (-7.84, -0.42) | 0.03 |
| Age | 0.04 (-0.08, 0.17) | 0.51 |
| Gender (male) | -3.45 (-6.57, -0.34) | 0.03 |
| Time × HTN groups | 0.34 (-0.30, 0.93) | 0.32 |

*Abbreviations: LB, lower bound; UB, upper bound; OCT, optical coherence tomography; T2DM, type 2 diabetes mellitus; HTN: hypertension; CMT: central macular thickness; RNFL: retinal nerve fiber layer*

**Table E4-2.** Group effects on the OCT values at different time points (1:1, SNUBH, T2DM vs control cohorts)

|  | **Time period** | **T2DM group** | | |  | **Non-T2DM group** | | | ***P* value** |
| --- | --- | --- | --- | --- | --- | --- | --- | --- | --- |
|  |  | **Expected value** | **LB** | **UB** |  | **Expected value** | **LB** | **UB** |  |
| **CMT** | 0 year | 271 | 258 | 285 |  | 275 | 268 | 283 | 0.59 |
|  | 5 years | 267 | 255 | 279 |  | 285 | 265 | 305 | 0.12 |
|  | 10 years | 263 | 243 | 283 |  | 294 | 256 | 333 | 0.15 |
|  | 15 years | 258 | 227 | 289 |  | 303 | 246 | 361 | 0.17 |
| **RNFL** | 0 year | 89.4 | 86.9 | 91.8 |  | 89.0 | 86.7 | 91.2 | 0.80 |
|  | 5 years | 87.8 | 85.4 | 90.2 |  | 87.6 | 85.3 | 89.9 | 0.91 |
|  | 10 years | 86.2 | 83.4 | 89.0 |  | 86.3 | 83.5 | 89.0 | 0.98 |
|  | 15 years | 84.6 | 81.1 | 88.1 |  | 84.9 | 81.5 | 88.4 | 0.90 |

Results are averaged over the levels of age and gender. *Abbreviations: LB, lower bound; UB, upper bound; OCT, optical coherence tomography; T2DM, type 2 diabetes mellitus; CMT: central macular thickness; RNFL: retinal nerve fiber layer*

**Table E4-3.** Group effects on the OCT values at different time points (1:1, SNUBH, HTN vs control cohorts)

|  | **Time period** | **HTN group** | | |  | **Non-HTN group** | | | ***P* value** |
| --- | --- | --- | --- | --- | --- | --- | --- | --- | --- |
|  |  | **Expected value** | **LB** | **UB** |  | **Expected value** | **LB** | **UB** |  |
| **CMT** | 0 year | 269 | 258 | 280 |  | 272 | 267 | 276 | 0.64 |
|  | 5 years | 266 | 259 | 274 |  | 264 | 248 | 280 | 0.83 |
|  | 10 years | 264 | 252 | 275 |  | 257 | 225 | 290 | 0.70 |
|  | 15 years | 261 | 243 | 280 |  | 250 | 201 | 299 | 0.67 |
| **RNFL** | 0 year | 89.8 | 86.7 | 92.9 |  | 93.9 | 91.9 | 96.0 | 0.03 |
|  | 5 years | 87.5 | 84.9 | 90.1 |  | 90.1 | 87.3 | 92.9 | 0.18 |
|  | 10 years | 85.2 | 81.7 | 88.7 |  | 86.2 | 81.5 | 91.0 | 0.73 |
|  | 15 years | 82.9 | 77.8 | 88.1 |  | 82.4 | 75.4 | 89.4 | 0.91 |

Results are averaged over the levels of age and gender. *Abbreviations: LB, lower bound; UB, upper bound; OCT, optical coherence tomography; HTN, hypertension; CMT: central macular thickness; RNFL: retinal nerve fiber layer*

**Table E4-4.** A mixed-effects regression analysis of OCT values (1:2, SNUBH)

| **Fixed effect** | **Estimate, β (95% CI)** | ***P* value** |
| --- | --- | --- |
| T2DM and non-T2DM groups | | |
| CMT | | |
| Time | 6.99 (3.86, 10.13) | <0.01 |
| T2DM groups | -1.69 (-16.60, 13.21) | 0.824 |
| Age | -0.11 (-0.50, 0.27) | 0.563 |
| Gender (male) | 6.55 (-4.05, 17.15) | 0.225 |
| Time × T2DM groups | -7.78 (-11.84, -3.71) | <0.01 |
| RNFL | | |
| Time | -0.43 (-0.60, -0.26) | <0.01 |
| T2DM groups | 0.04 (-2.89, 2.98) | 0.98 |
| Age | -0.18 (-0.29, -0.08) | <0.01 |
| Gender (male) | -1.37 (-4.13, -1.40) | 0.33 |
| Time × T2DM groups | 0.10 (-0.13, 0.32) | 0.39 |
| HTN and non-HTN groups | | |
| CMT | | |
| Time | -1.61 (-4.65, 1.42) | 0.30 |
| HTN groups | -3.82 (-16.30, 8.67) | 0.55 |
| Age | 0.04 (-0.24, 0.33) | 0.76 |
| Gender (male) | 8.50 (1.55, 15.45) | 0.02 |
| Time × HTN groups | 1.04 (-2.49, 4.56) | 0.56 |
| RNFL | | |
| Time | -0.54 (-1.00, -0.09) | 0.02 |
| HTN groups | -2.87 (-6.52, 0.79) | 0.12 |
| Age | 0.02 (-0.09, 0.12) | 0.74 |
| Gender (male) | -3.50 (-6.15, -0.86) | <0.01 |
| Time × HTN groups | 0.15 (-0.49, 0.78) | 0.65 |

*Abbreviations: LB, lower bound; UB, upper bound; OCT, optical coherence tomography; T2DM, type 2 diabetes mellitus; HTN: hypertension; CMT: central macular thickness; RNFL: retinal nerve fiber layer*

**Table E4-5.** Group effects on the OCT values at different time points (1:2, SNUBH, T2DM vs control cohorts)

|  | **Time period** | **T2DM group** | | |  | **Non-T2DM group** | | | ***P* value** |
| --- | --- | --- | --- | --- | --- | --- | --- | --- | --- |
|  |  | **Expected value** | **LB** | **UB** |  | **Expected value** | **LB** | **UB** |  |
| **CMT** | 0 year | 272 | 258 | 285 |  | 273 | 267 | 279 | 0.82 |
|  | 5 years | 268 | 256 | 279 |  | 308 | 292 | 324 | <0.01 |
|  | 10 years | 264 | 243 | 284 |  | 343 | 312 | 375 | <0.01 |
|  | 15 years | 260 | 227 | 292 |  | 378 | 331 | 425 | <0.01 |
| **RNFL** | 0 year | 89.4 | 87.0 | 91.9 |  | 89.4 | 87.7 | 91.1 | 0.54 |
|  | 5 years | 87.8 | 85.4 | 90.2 |  | 87.2 | 85.5 | 89.0 | 0.71 |
|  | 10 years | 86.1 | 83.4 | 88.9 |  | 85.1 | 82.9 | 87.3 | 0.54 |
|  | 15 years | 84.5 | 81.0 | 87.9 |  | 82.9 | 80.1 | 85.7 | 0.45 |

Results are averaged over the levels of age and gender. *Abbreviations: LB, lower bound; UB, upper bound; OCT, optical coherence tomography; T2DM, type 2 diabetes mellitus; CMT: central macular thickness; RNFL: retinal nerve fiber layer*

**Table E4-6.**Group effects on the OCT values at different time points (1:2, SNUBH, HTN vs control cohorts)

|  | **Time period** | **HTN group** | | |  | **Non-HTN group** | | | ***P* value** |
| --- | --- | --- | --- | --- | --- | --- | --- | --- | --- |
|  |  | **Expected value** | **LB** | **UB** |  | **Expected value** | **LB** | **UB** |  |
| **CMT** | 0 year | 269 | 257 | 280 |  | 272 | 268 | 276 | 0.55 |
|  | 5 years | 266 | 258 | 274 |  | 264 | 250 | 279 | 0.87 |
|  | 10 years | 263 | 250 | 275 |  | 256 | 227 | 286 | 0.69 |
|  | 15 years | 260 | 239 | 280 |  | 248 | 203 | 293 | 0.64 |
| **RNFL** | 0 year | 89.3 | 86.0 | 92.6 |  | 92.2 | 90.6 | 93.8 | 0.12 |
|  | 5 years | 87.3 | 84.8 | 89.8 |  | 89.4 | 87.0 | 91.8 | 0.23 |
|  | 10 years | 85.3 | 81.7 | 88.9 |  | 86.7 | 82.3 | 91.1 | 0.62 |
|  | 15 years | 83.3 | 77.8 | 88.8 |  | 84.0 | 77.4 | 90.6 | 0.87 |

Results are averaged over the levels of age and gender. *Abbreviations: LB, lower bound; UB, upper bound; OCT, optical coherence tomography; HTN, hypertension; CMT: central macular thickness; RNFL: retinal nerve fiber layer*

**Table E4-7.** A mixed-effects regression analysis of OCT values (1:4, SNUBH)

| **Fixed effect** | **Estimate, β (95% CI)** | ***P* value** |
| --- | --- | --- |
| T2DM and non-T2DM groups | | |
| CMT | | |
| Time | 3.57 (1.58, 5.55) | <0.01 |
| T2DM groups | -1.40 (-14.93, 12.14) | 0.84 |
| Age | -0.08 (-0.35, 0.19) | 0.57 |
| Gender (male) | 6.87 (-0.87, 14.62) | 0.08 |
| Time × T2DM groups | -4.35 (-7.57, -1.14) | <0.01 |
| RNFL | | |
| Time | -0.62 (-0.79, -0.46) | <0.01 |
| T2DM groups | -0.98 (-3.76, 1.81) | 0.49 |
| Age | -0.11 (-0.19, -0.03) | <0.01 |
| Gender (male) | -2.63 (-4.85, -0.42) | 0.02 |
| Time × T2DM groups | -0.25 (-0.04, 0.53) | 0.09 |
| HTN and non-HTN groups | | |
| CMT | | |
| Time | -1.44 (-4.27, 1.39) | 0.32 |
| HTN groups | -3.79 (-18.00, 10.43) | 0.60 |
| Age | 0.00 (-0.26, 0.27) | 0.97 |
| Gender (male) | 12.20 (5.27, 19.14) | <0.01 |
| Time × HTN groups | 0.82 (-2.70, 4.33) | 0.65 |
| RNFL | | |
| Time | -0.79 (-1.11, -0.46) | <0.01 |
| HTN groups | -1.61 (-4.91, 1.68) | 0.34 |
| Age | 0.03 (-0.05, 0.11) | 0.46 |
| Gender (male) | -3.50 (-5.62, -1.38) | <0.01 |
| Time × HTN groups | 0.36 (-0.16, 0.88) | 0.17 |

*Abbreviations: LB, lower bound; UB, upper bound; OCT, optical coherence tomography; T2DM, type 2 diabetes mellitus; HTN: hypertension; CMT: central macular thickness; RNFL: retinal nerve fiber layer*

**Table E4-8.**Group effects on the OCT values at different time points (1:4, SNUBH, T2DM vs control cohorts)

|  | **Time period** | **T2DM group** | | |  | **Non-T2DM group** | | | ***P* value** |
| --- | --- | --- | --- | --- | --- | --- | --- | --- | --- |
|  |  | **Expected value** | **LB** | **UB** |  | **Expected value** | **LB** | **UB** |  |
| **CMT** | 0 year | 271 | 258 | 284 |  | 273 | 269 | 277 | 0.84 |
|  | 5 years | 268 | 257 | 278 |  | 291 | 280 | 301 | <0.01 |
|  | 10 years | 264 | 244 | 283 |  | 309 | 289 | 328 | <0.01 |
|  | 15 years | 260 | 228 | 291 |  | 326 | 297 | 356 | <0.01 |
| **RNFL** | 0 year | 89.6 | 87.1 | 92.2 |  | 90.6 | 89.4 | 91.9 | 0.49 |
|  | 5 years | 87.8 | 85.4 | 90.1 |  | 87.5 | 86.1 | 88.9 | 0.85 |
|  | 10 years | 85.9 | 83.0 | 88.8 |  | 84.4 | 82.5 | 86.3 | 0.37 |
|  | 15 years | 84.0 | 80.3 | 87.8 |  | 81.3 | 78.7 | 83.9 | 0.21 |

Results are averaged over the levels of age and gender. *Abbreviations: LB, lower bound; UB, upper bound; OCT, optical coherence tomography; T2DM, type 2 diabetes mellitus; CMT: central macular thickness; RNFL: retinal nerve fiber layer*

**Table E4-9.** Group effects on the OCT values at different time points (1:4, SNUBH, HTN vs control cohorts)

|  | **Time period** | **HTN group** | | |  | **Non-HTN group** | | | ***P* value** |
| --- | --- | --- | --- | --- | --- | --- | --- | --- | --- |
|  |  | **Expected value** | **LB** | **UB** |  | **Expected value** | **LB** | **UB** |  |
| **CMT** | 0 year | 268 | 255 | 282 |  | 272 | 268 | 276 | 0.60 |
|  | 5 years | 265 | 256 | 275 |  | 265 | 251 | 279 | 0.97 |
|  | 10 years | 262 | 248 | 277 |  | 258 | 230 | 286 | 0.78 |
|  | 15 years | 259 | 236 | 283 |  | 251 | 209 | 293 | 0.73 |
| **RNFL** | 0 year | 89.5 | 86.4 | 92.5 |  | 91.1 | 89.9 | 92.3 | 0.34 |
|  | 5 years | 87.3 | 84.9 | 89.8 |  | 87.2 | 85.4 | 88.9 | 0.90 |
|  | 10 years | 85.2 | 81.9 | 88.6 |  | 83.2 | 80.1 | 86.4 | 0.39 |
|  | 15 years | 83.1 | 78.1 | 88.1 |  | 79.3 | 74.6 | 84.0 | 0.27 |

Results are averaged over the levels of age and gender. *Abbreviations: LB, lower bound; UB, upper bound; OCT, optical coherence tomography; HTN, hypertension; CMT: central macular thickness; RNFL: retinal nerve fiber layer*

**Figure E1.** Cohort definitions for HTN and control groups. A) Patients with HTN, excluding those diagnosed or treated for choroidal or retinal disorders, who have undergone OCT imaging. B) Patients without HTN or any choroidal or retinal disorders, who have undergone OCT imaging. *Abbreviations: HTN: hypertension; OCT: optical coherence tomography*


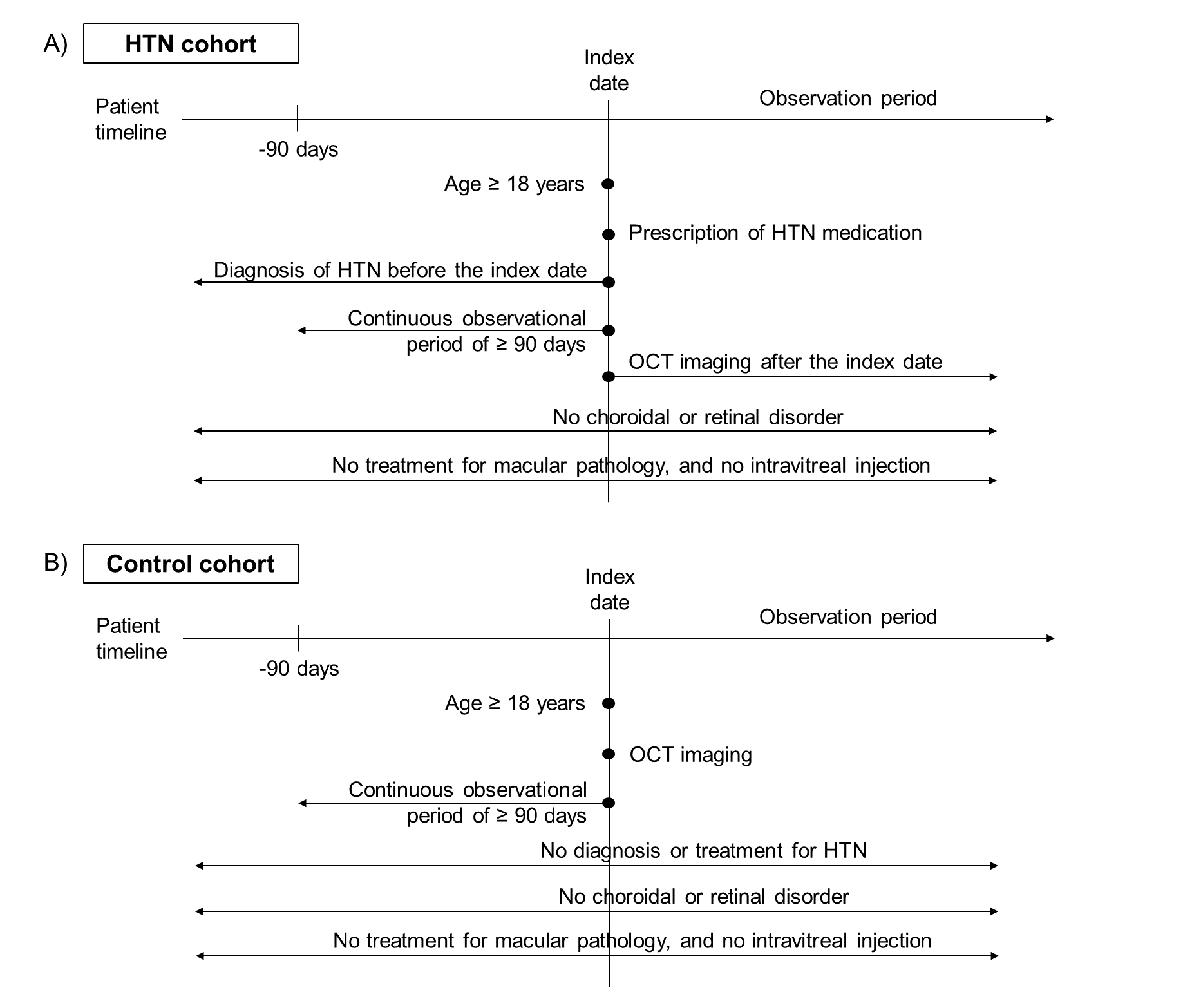


**Figure E2-1.** Flowchart of the study population comparing T2DM and control cohorts at AUMC. *Abbreviations:* T2DM*: type 2 diabetes mellitus; OCT: optical coherence tomography*


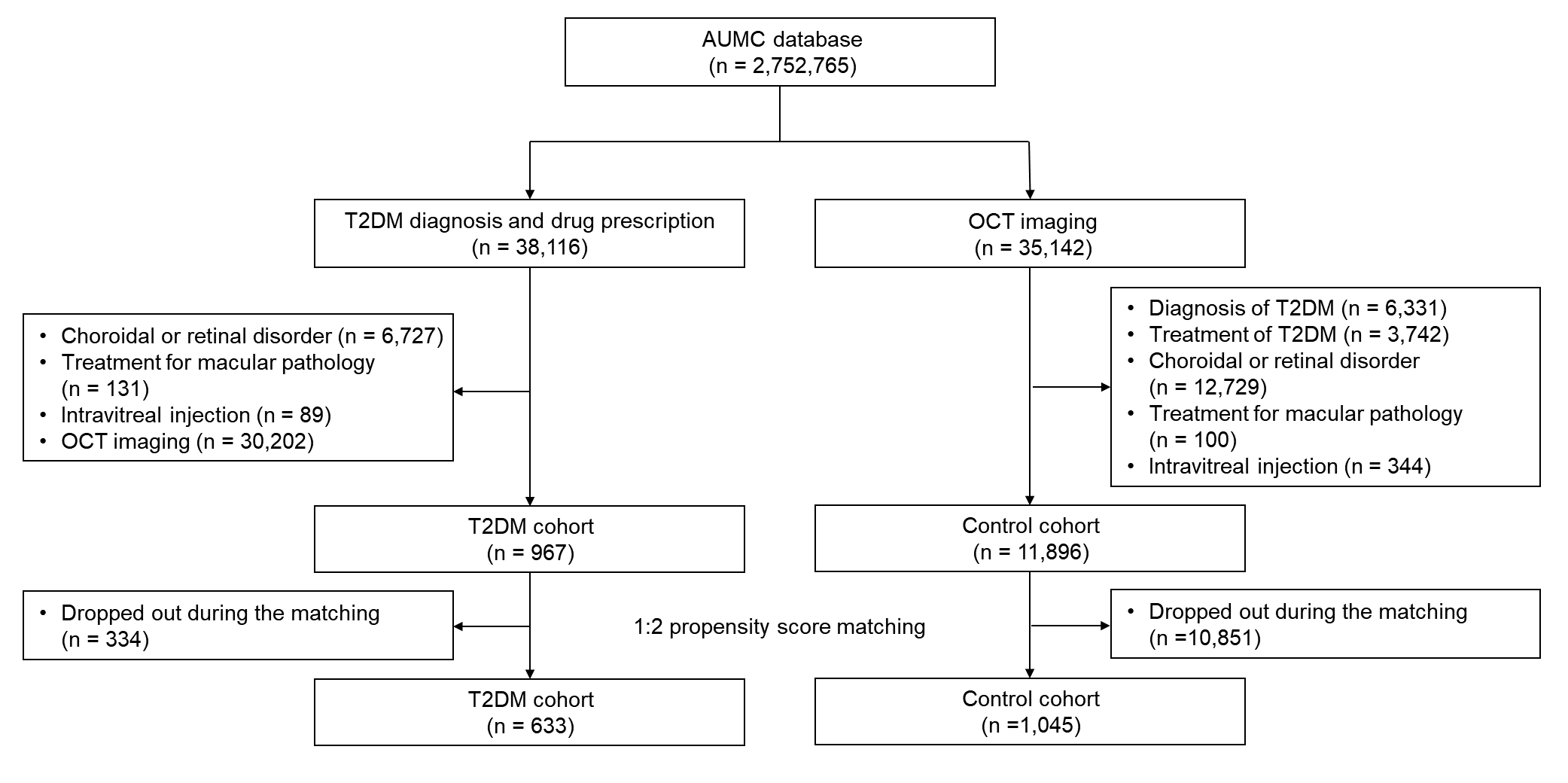


**Figure E2-2.** Flowchart of the study population comparing T2DM and control cohorts at SNUBH. *Abbreviations:* T2DM*: type 2 diabetes mellitus; OCT: optical coherence tomography*


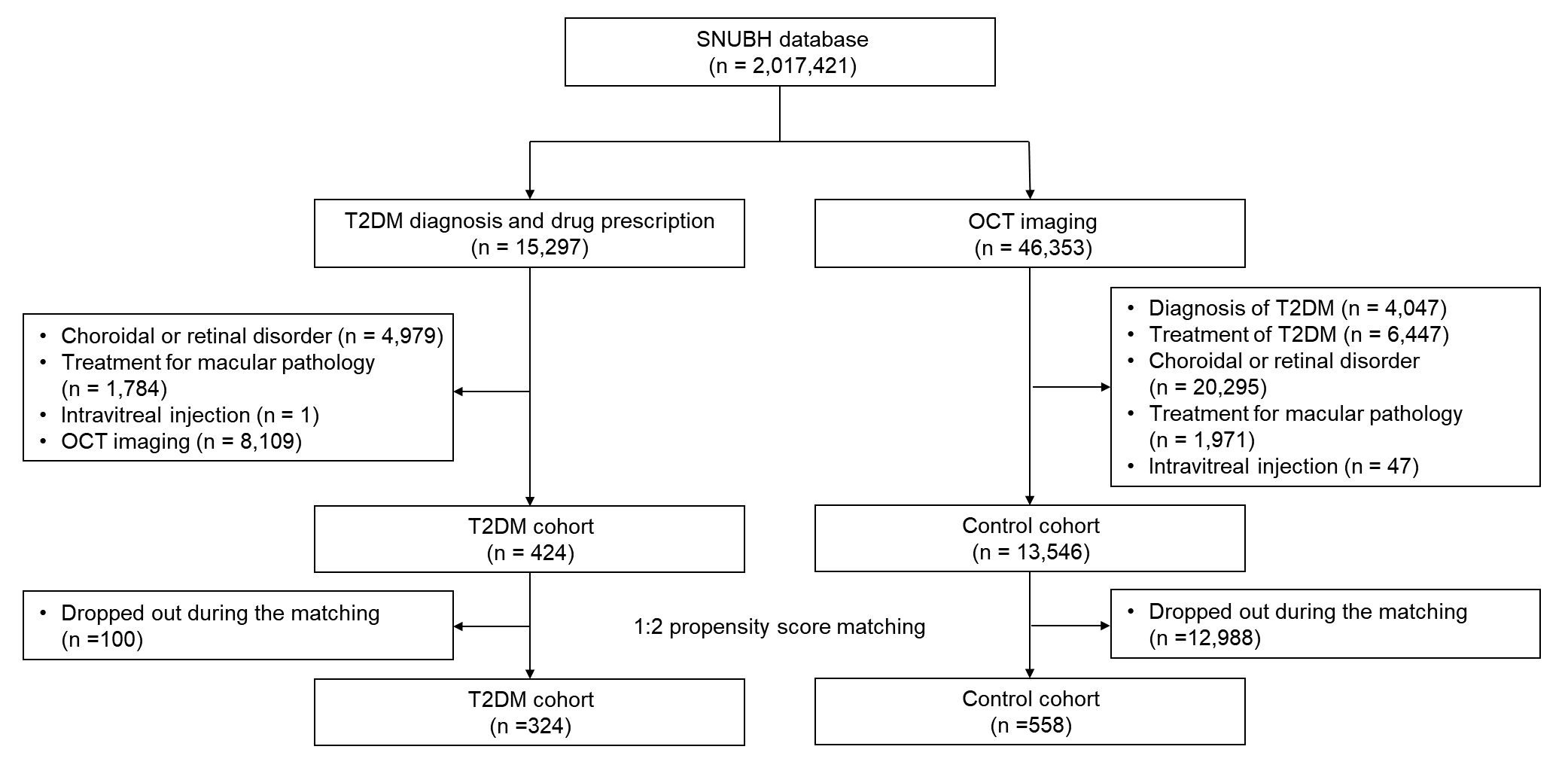


**Figure E3-1.** Flowchart of the study population comparing HTN and control cohorts. *Abbreviations:* HTN*: hypertension; OCT: optical coherence tomography*


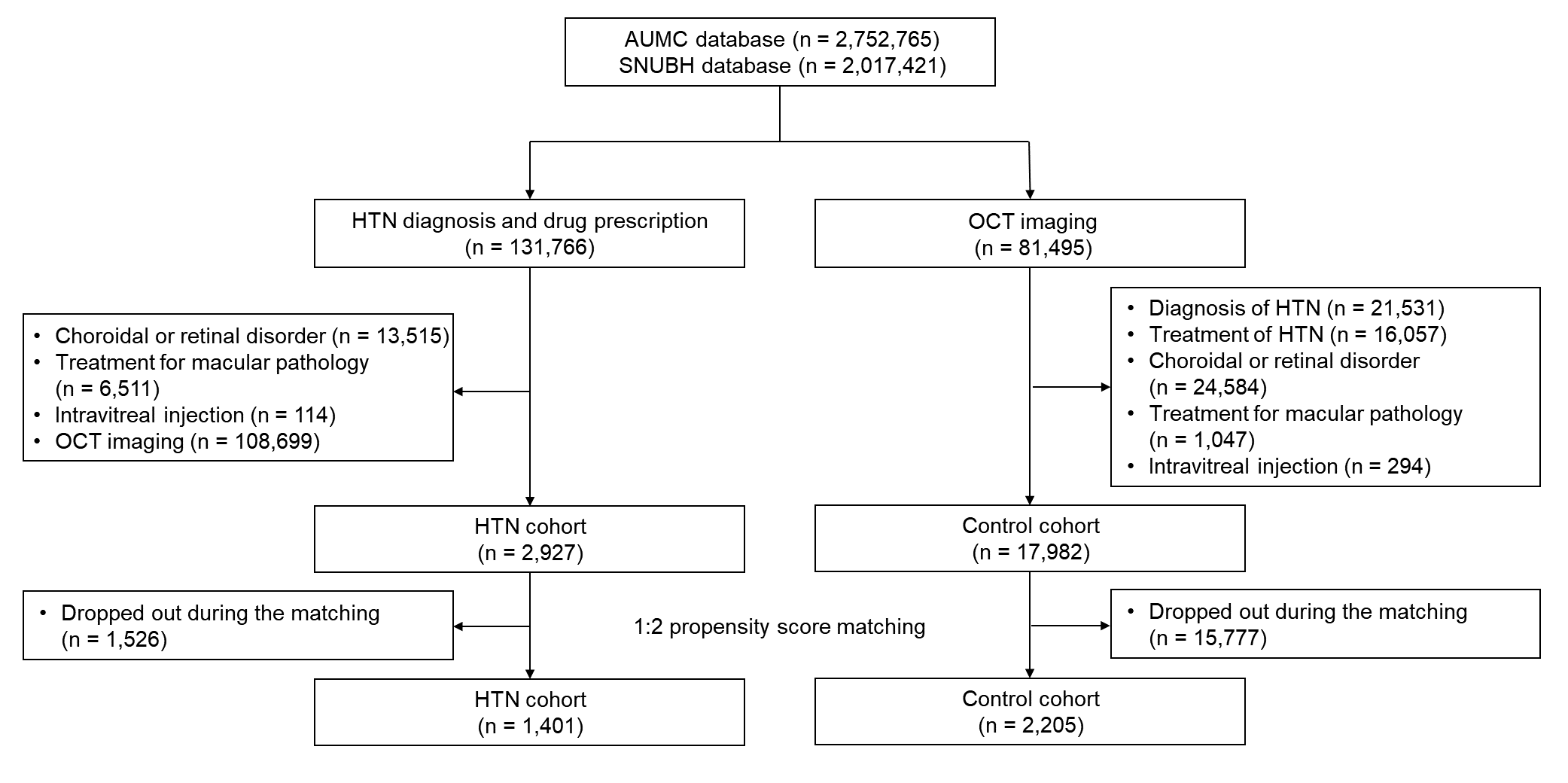


**Figure E3-2.** Flowchart of the study population comparing HTN and control cohorts at AUMC. *Abbreviations:* HTN*: hypertension; OCT: optical coherence tomography*


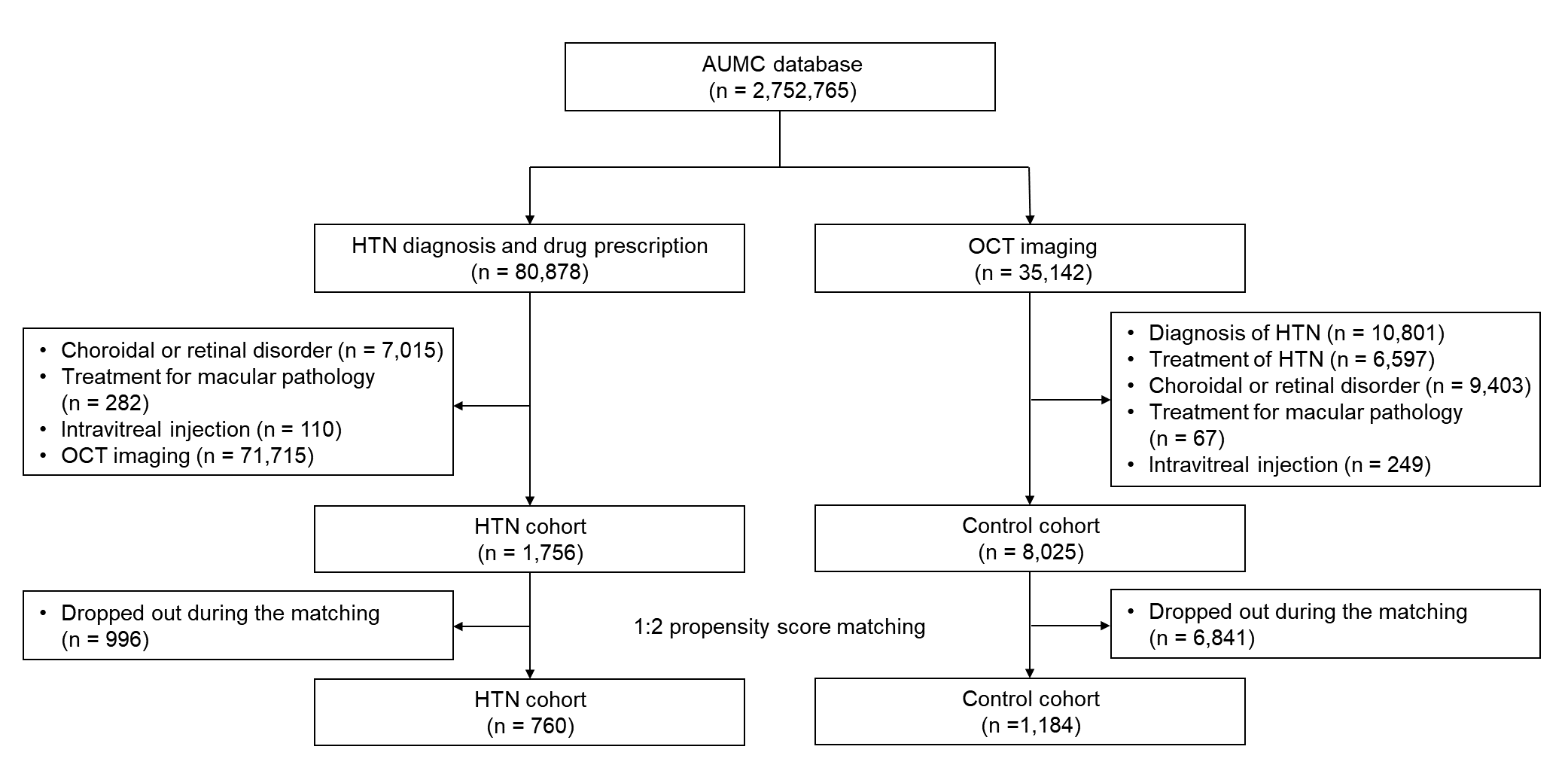


**Figure E3-3.** Flowchart of the study population comparing HTN and control cohorts at SNUBH. *Abbreviations:* HTN*: hypertension; OCT: optical coherence tomography*

*
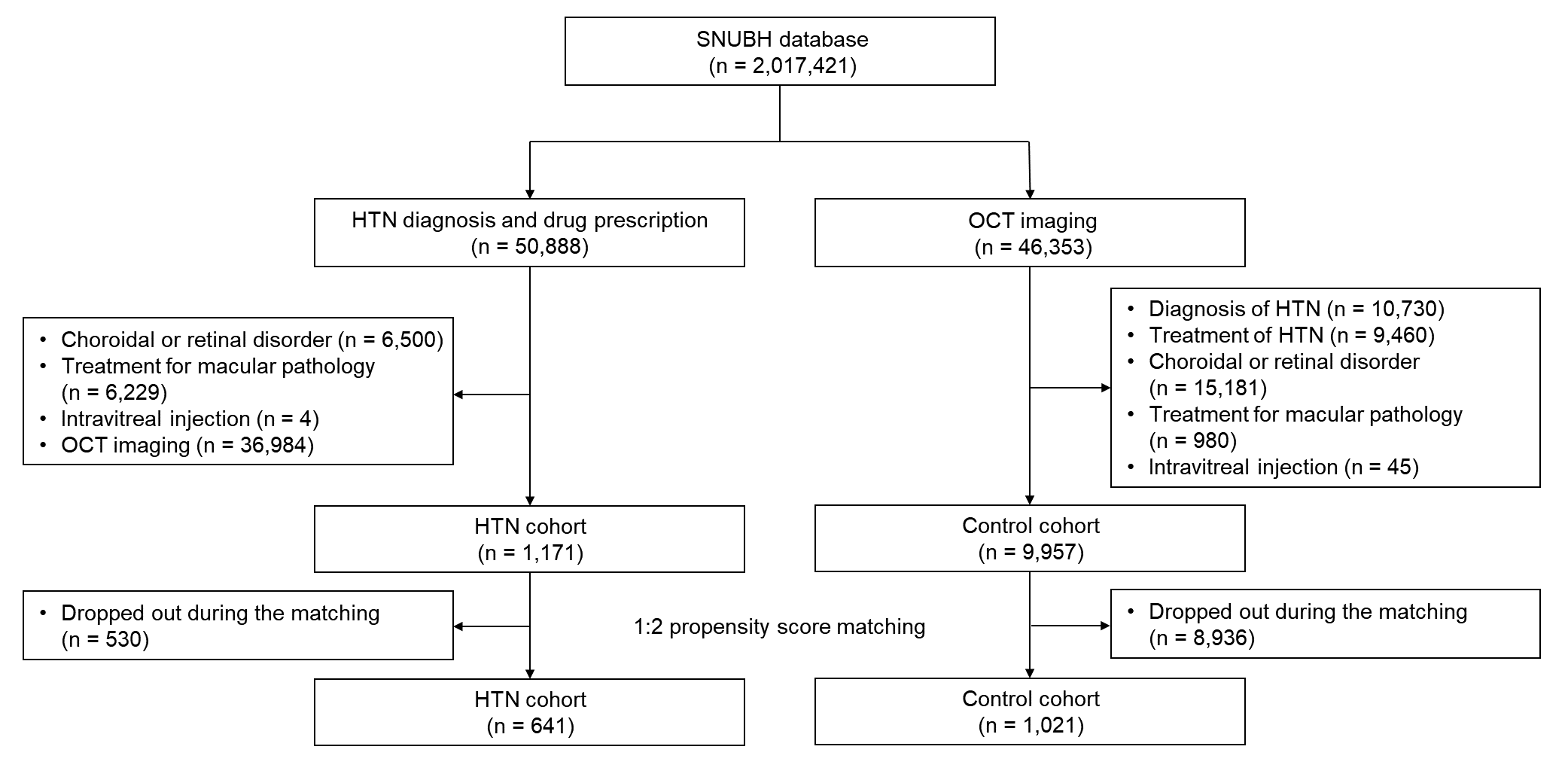
*

**Figure E4-1.** Longitudinal analysis of laboratory values using a linear mixed effects model after 1:1 propensity score matching of T2DM and control cohorts. A) Central macular thickness at AUMC; B) Central macular thickness at SNUBH; C) RNFL thickness at AUMC; D) RNFL thickness at SNUBH. Abbreviations: T2DM: type 2 diabetes mellitus; RNFL: retinal nerve fiber layer


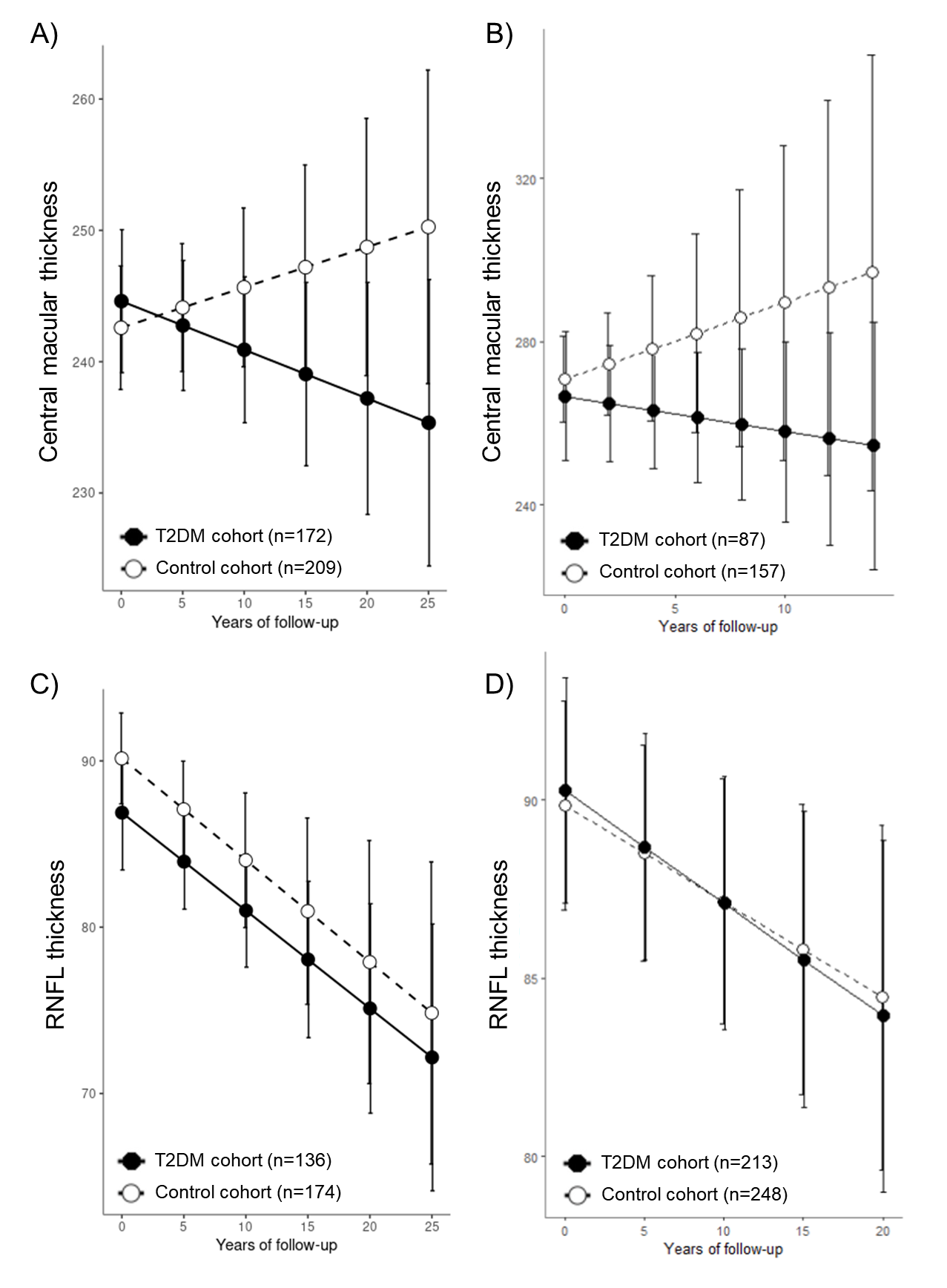


**Figure E4-2.** Longitudinal analysis of laboratory values using a linear mixed effects model after 1:4 propensity score matching of T2DM and control cohorts. A) Central macular thickness at AUMC; B) Central macular thickness at SNUBH; C) RNFL thickness at AUMC; D) RNFL thickness at SNUBH. Abbreviations: T2DM: type 2 diabetes mellitus; RNFL: retinal nerve fiber layer


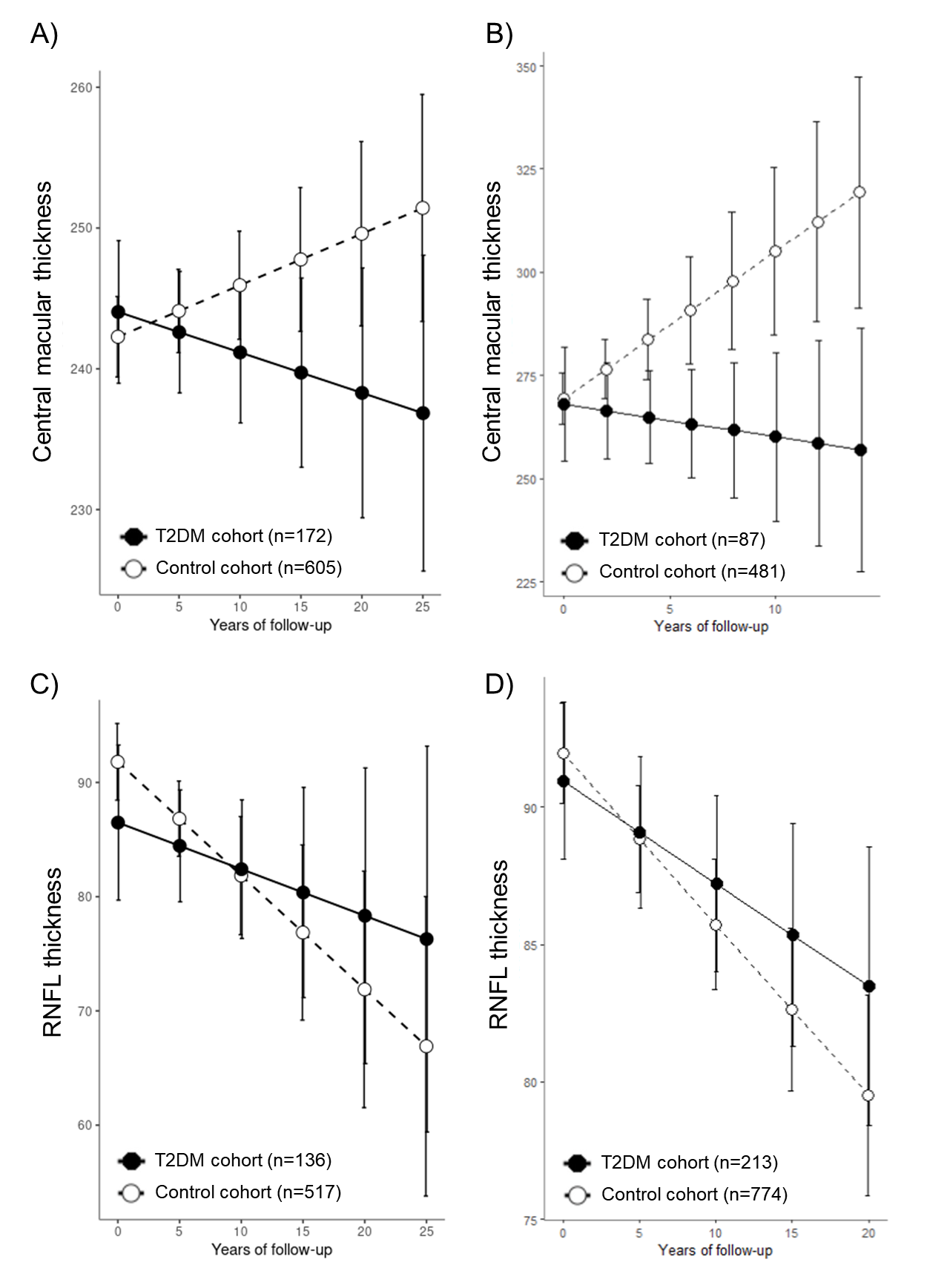


**Figure E5-1.** Longitudinal analysis of laboratory values using a linear mixed effects model after 1:1 propensity score matching of HTN and control cohorts. A) Central macular thickness at AUMC; B) Central macular thickness at SNUBH; C) RNFL thickness at AUMC; D) RNFL thickness at SNUBH. Abbreviations: HTN: hypertension; RNFL: retinal nerve fiber layer


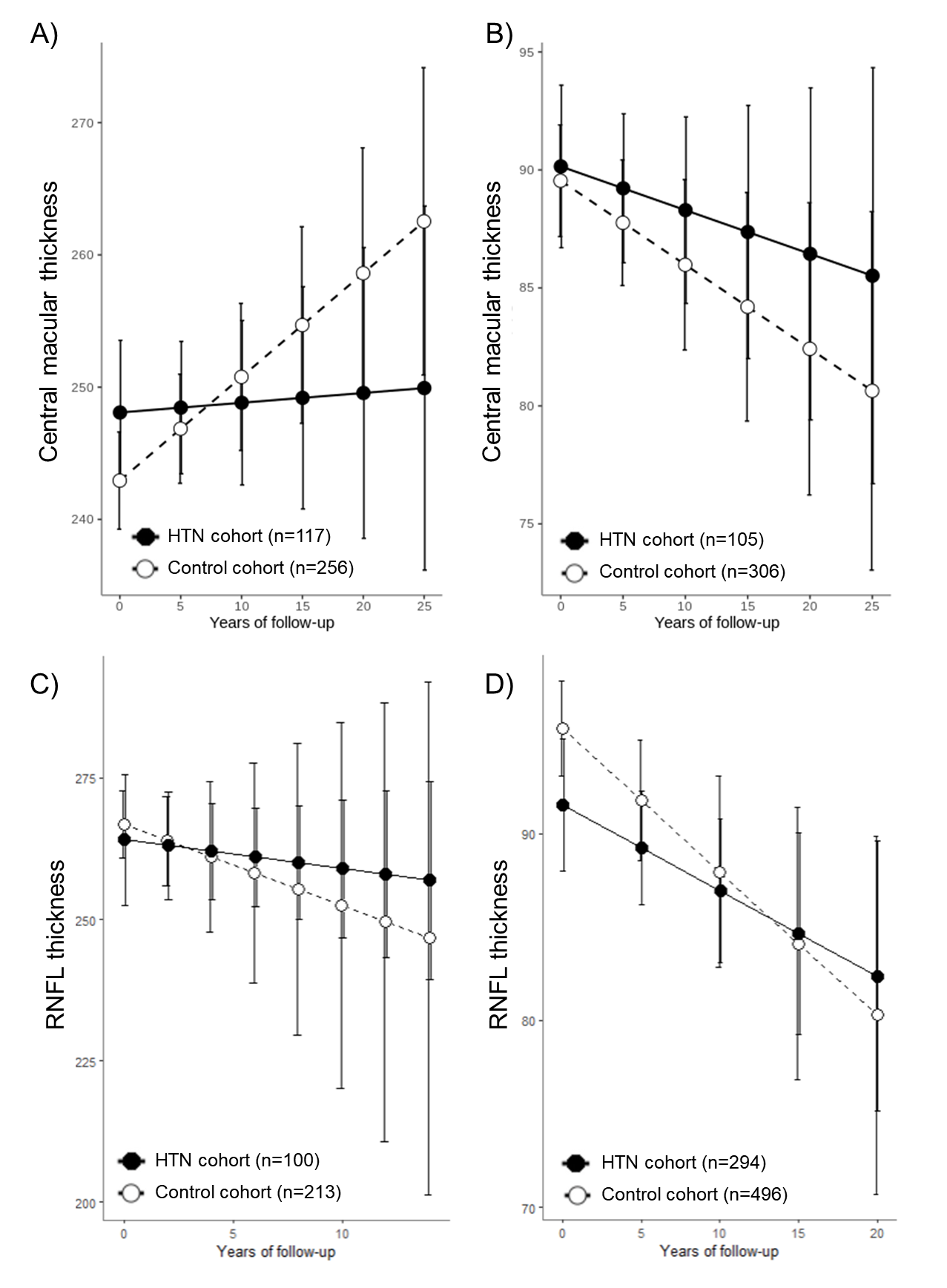


**Figure E5-2.** Longitudinal analysis of laboratory values using a linear mixed effects model after 1:2 propensity score matching of HTN and control cohorts. A) Central macular thickness at AUMC; B) Central macular thickness at SNUBH; C) RNFL thickness at AUMC; D) RNFL thickness at SNUBH. Abbreviations: HTN: hypertension; RNFL: retinal nerve fiber layer


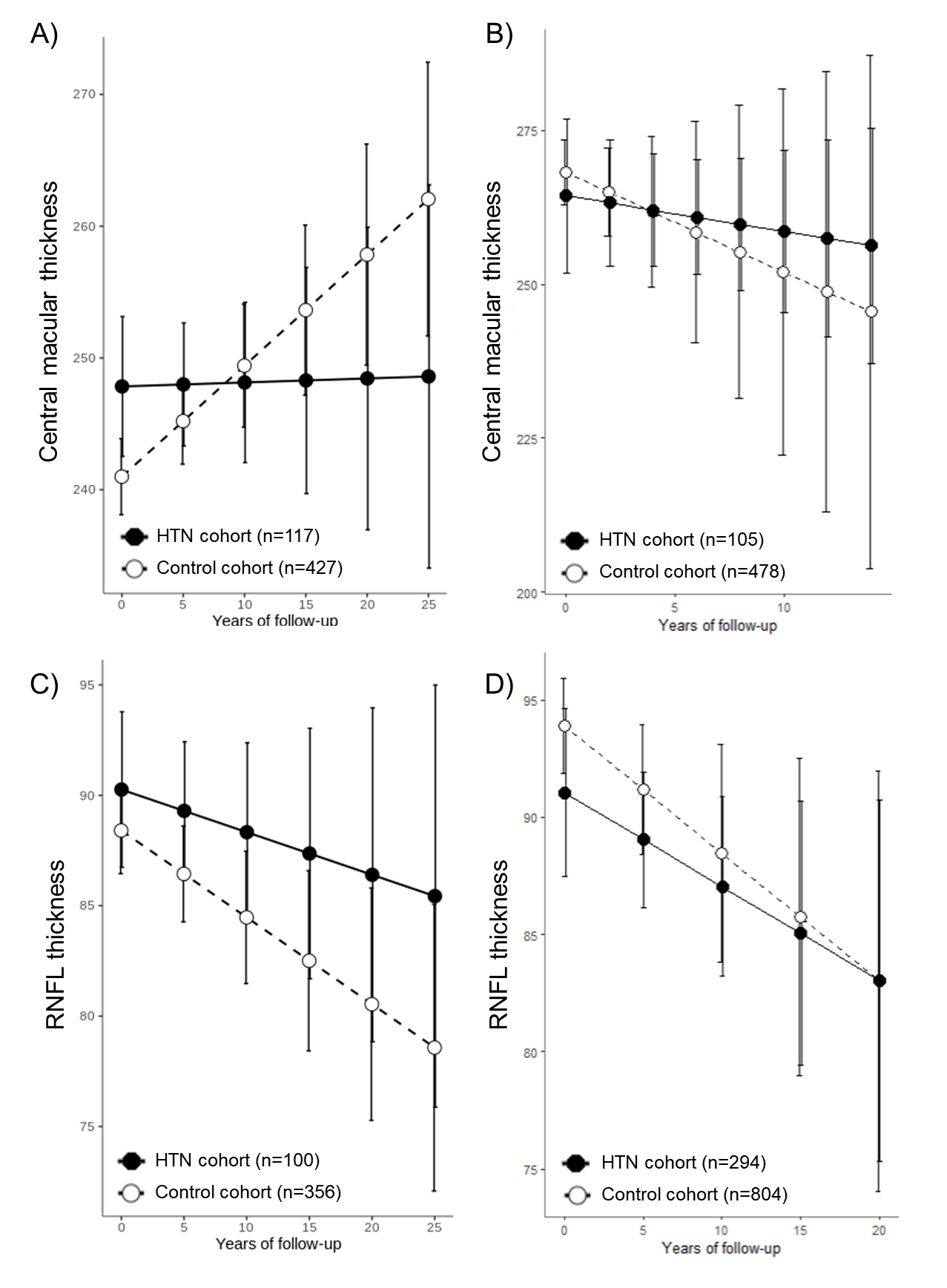


**Figure E5-3.** Longitudinal analysis of laboratory values using a linear mixed effects model after 1:4 propensity score matching of HTN and control cohorts. A) Central macular thickness at AUMC; B) Central macular thickness at SNUBH; C) RNFL thickness at AUMC; D) RNFL thickness at SNUBH. Abbreviations: HTN: hypertension; RNFL: retinal nerve fiber layer


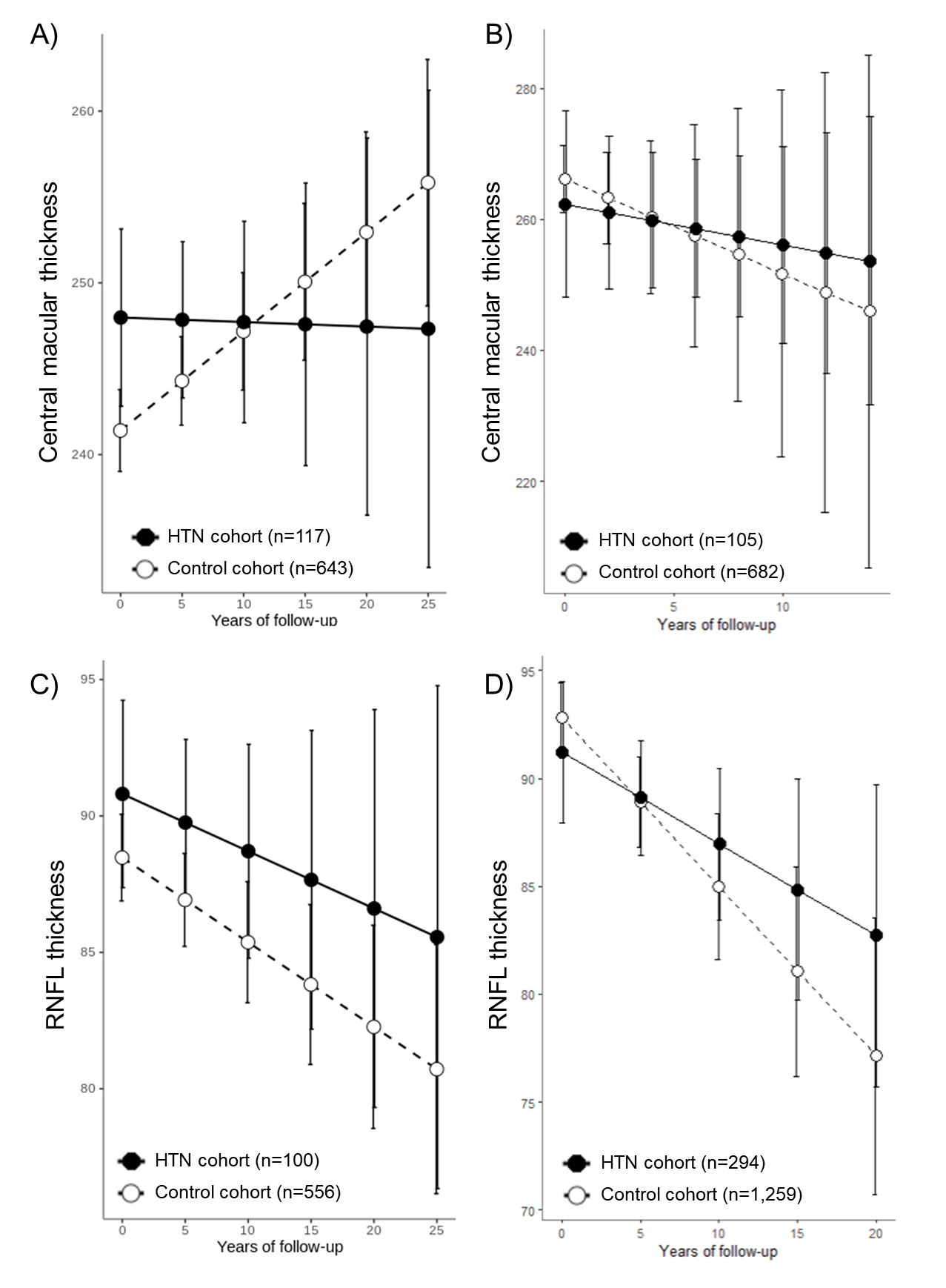

Supplement: Multimedia Appendix 1 [file medinform-v13-e64422-s001.docx]
